# Ionically Counter‐Programmable Phase‐Separation of Superstable Star‐Biopolyamide Coacervates for Electrothermally‐Responsive Pseudosolid Smart Windows

**DOI:** 10.1002/advs.78227

**Published:** 2026-10-11

**Authors:** Jie Liu, Xiaotian Zhu, Jinjian Hou, Jinze Du, Mohammad Asif Ali, Maiko K. Okajima, Tatsuo Kaneko

**Affiliations:** ^1^ The Key Laboratory of Synthetic and Biological Colloids School of Chemical and Material Engineering Ministry of Education Jiangnan University Wuxi China; ^2^ School of Chemistry and Chemical Engineering Hainan University Haikou China; ^3^ School of Chemical Engineering Tianjin University Tianjin China; ^4^ Zhejiang Institute of Tianjin University Ningbo China

**Keywords:** coacervation, coordination, electrothermal response, pseudosolid, salting‐in effect, smart window, star‐branch, thermoresponsive polymers

## Abstract

Thermoresponsive polymers are highly anticipated for passive thermochromic smart windows, yet the instability and too‐high mobility of their light‐scattering states for separated phases remain significant hurdles. To address these challenges, we report a design concept for pyrrolidone‐based star‐polyamides synthesized from itaconic acid and cadaverine, whose architecture provides exceptional sensitivity to external stimuli. In a stark departure from the classical Hofmeister series, Ca^2+^ initiates an anomalous salting‐in effect that contrasts with the Na^+^‐induced salting‐out. DFT/MD calculations reveal that pyrrolidone moderately coordinates with Ca^2+^ without disrupting its hydration shell, thereby imparting a dense positive charge to the coacervate surfaces. Crucially, the cationic surface charge endows the coacervates with light‐scattering bodies with superior dispersion stability, surpassing conventional PNIPAM by over 20‐fold. The system maintains robust stability at 5 mol/L, a regime where conventional thermoresponsive polymers inevitably fail. An applied electric field enables spatial control of transparency, creating a dual electro‐thermal responsive system for energy‐efficient, sustainable smart windows. Furthermore, the Ca^2+^ salting‐in effects enable coacervation in a high polymer concentration range over 50% to establish pseudosolidity, which provides a versatile platform for next‐generation electro‐thermal soft systems, ranging from implantable biosensors to battery‐operated smart windows.

## Graphical abstract

We develop bio‐based, star‐branched polyamides from itaconic acid and cadaverine with tunable lower critical solution temperature (LCST) transitions for dynamic solar modulation. Specific metal ion coordination with pyrrolidone moieties drives precise phase transitions via calcium‐induced salting‐in and sodium‐induced salting‐out mechanisms. Formulated as ultra‐high‐concentration pseudo‐solids, these responsive polymers enable highly efficient, multi‐stimuli smart windows for advanced energy‐saving applications.

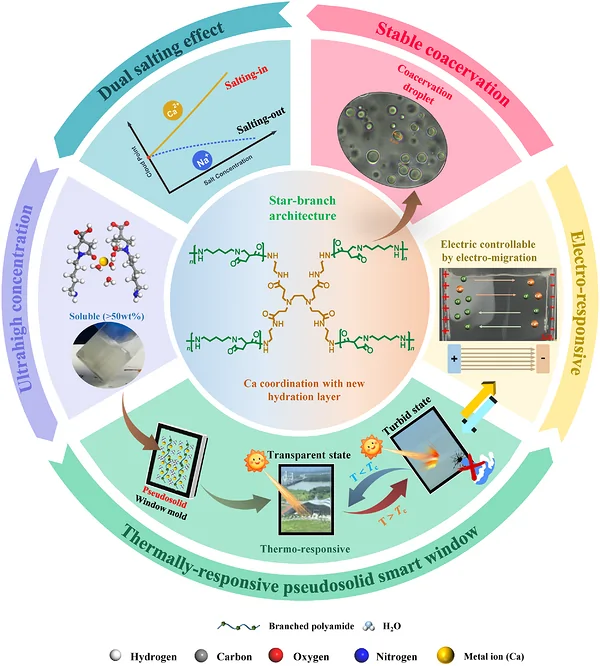

## 1 Introduction

The escalating global energy crisis underscores an urgent need for sustainable technologies, particularly in buildings, automobiles, and aircraft, where windows account for a disproportionate share of energy loss [1]. Thermochromic smart windows, which passively regulate solar irradiance in response to temperature, represent a promising solution for reducing heating and cooling loads [2, 3, 4]. In contrast, organic thermoresponsive polymers like poly(N‐isopropylacrylamide) (PNIPAM) [5], hydroxypropyl cellulose (HPC) [6], and poly(vinylmethylether) (PVME) [7] exhibit a lower critical solution temperature (LCST) and offer tunable switching near ambient conditions and facile processing [5, 8], differently from inorganic materials [9, 10] like vanadium dioxide (VO_2_) [4, 11, 12, 13, 14] and tungsten trioxide (WO_3_) [15, 16].

However, the widespread adoption of these polymers is hindered by two critical instabilities: 1) the dispersed light‐scattering domains tend to coalesce into a continuous macro‐layer within several tens of minutes; 2) the risk of solution leakage to damage device safety and long‐term operational validity [17, 18, 19]. Current strategies to mitigate this instability, such as forming polymer networks or incorporating nanoparticle stabilizers, introduce new compromises, including sluggish response kinetics, reduced optical transparency, process difficulty [20, 21, 22, 23], or long‐term compatibility issues [24]. Introducing the concept of pseudosolid [25] can elegantly resolve this issue. But conventional linear polymers inherently lead to severe restriction of polymer chain mobility and unacceptably sluggish stimuli‐responsiveness.

Based on this background, the water‐soluble polymers having a salting‐in property should be a good selection. However, even the solubility of popular water‐soluble ones like poly(ethylene glycol) (PEG) and polyvinylpyrrolidone (PVP) is severely compromised at high salt concentration [26]. This phenomenon, known as salting‐out, is governed by the classic Hofmeister series (e.g., NH_4_^+^ > K^+^ > Na^+^ > Cs^+^ > Li^+^ > Rb^+^ >Mg^2+^ > Ca^2+^ > Ba^2+^) [27, 28]. To realize this pseudosolid system, we directed our strategy toward polymers capable of achieving ultra‐high concentrations under specific external modulations, such as the introduction of salt ions. Fundamentally, the aqueous solubility of thermoresponsive polymers is a complex physicochemical property, dictated by a delicate equilibrium of polymer‐solvent interactions, polymer‐polymer interactions, and entropic factors. Consequently, designing and synthesizing polymers that can maintain high solubility under elevated ionic strength conditions remains a significant challenge, necessitating substantial further research.

Herein, inspired by the mild and dynamic metal‐coordination system of PVP [29, 30, 31, 32] widely applied in fabricating metal nanoparticles, semiconductor thin films, metal–organic frameworks, and quantum dots, we design amphiphilically balanced star‐polyamides featuring pyrrolidone backbones [33, 34] derived from itaconic acid and cadaverine. We found a unique phenomenon where the pyrrolidone moiety forms a mild coordination bond with calcium while preserving the pre‐existing calcium–water coordination, resulting in a distinct state where calcium is coordinated by both pyrrolidone and water. This dual affinity exerts a salting‐in effect that recruits water molecules around the polyamide matrix, driving several significant phenomena: 1) an increase in the LCST of the star‐polyamide solution, 2) super‐stabilization of the coacervate dispersion, 3) electrophoretically responsive coacervation, and 4) the realization of LCST behavior even at high concentrations (Scheme 1). Consequently, this star‐polyamide system overcomes the longstanding trade‐off between ionic stability and responsive functionality, paving the way for robust pseudo‐solid smart windows operable in complex environments, while demonstrating strong potential for self‐powered windows and bio‐integrated smart devices.

**SCHEME 1:**
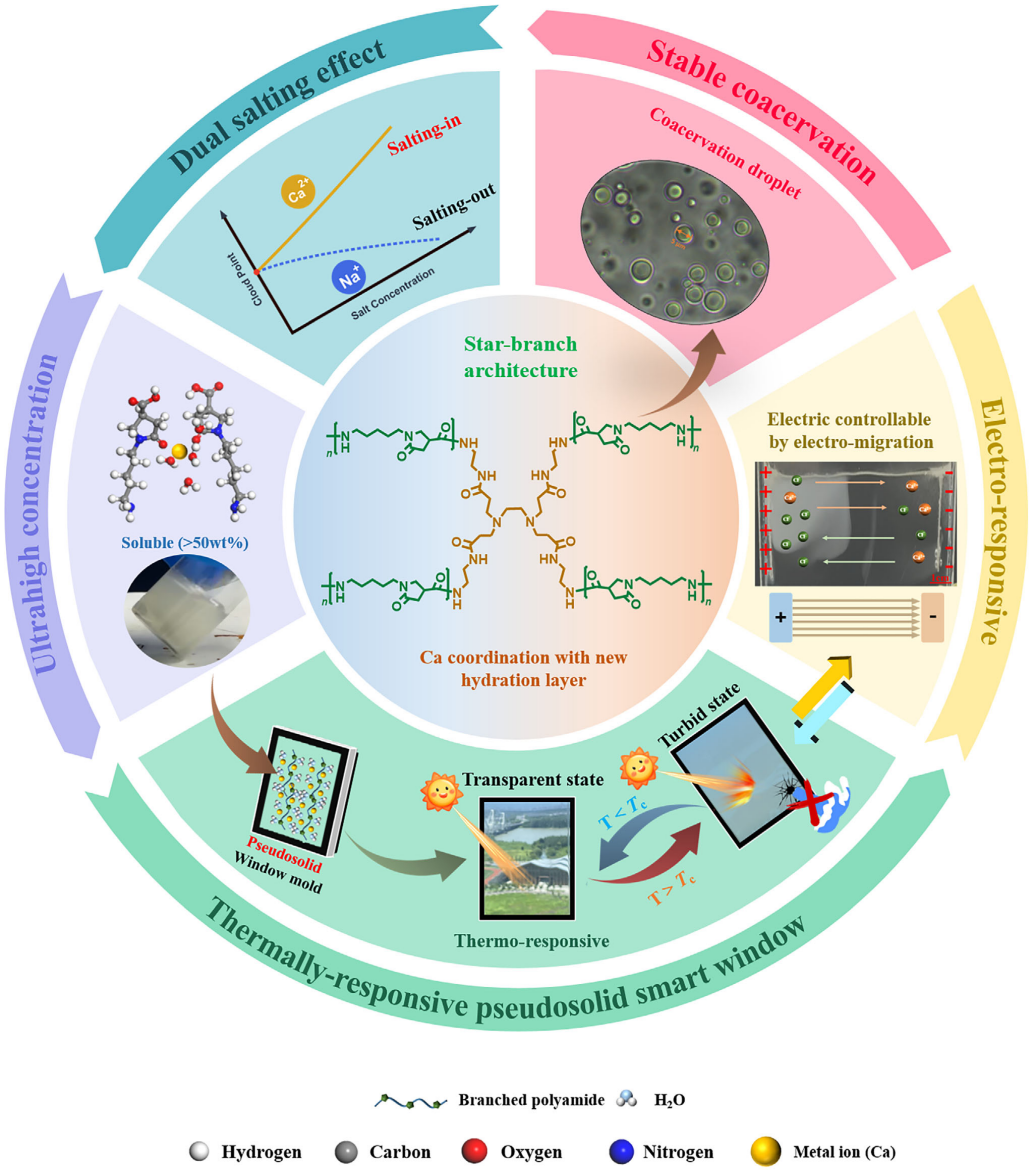
Design and advantages of thermochromic star‐polyamides. Anomalous salting‐in and unique coordination formation. Dual pseudosolid smart window design using star‐polyamides.

## 2 Results and Discussion

### 2.1 Design and Synthesis of Star‐Polyamides

The manifestation of LCST phase behavior requires a precise hydrophilic‐hydrophobic balance, which can be engineered into water‐soluble polymers (e.g., PNIPAM) [5, 35, 36] or induced in naturally hydrophilic polymers (e.g., HPC) [6, 37, 38, 39, 40, 41, 42]. In our previous work, we developed high‐performance bio‐based polyamides from itaconic acid (IA), demonstrating excellent thermomechanical properties and biocompatibility [33, 34]. However, their water solubility was either too high or too low for thermoresponsive applications. We postulated that the intrinsic hydrophilic‐hydrophobic equilibrium of these pyrrolidone‐based polymers could be systematically tuned by varying the alkyl chain length of the diamine comonomer to achieve thermochromism. To test this, we synthesized a series of polyamides from itaconic acid and aliphatic diamines of different chain lengths (4, 5, and 6 carbons). However, these linear polymers failed to exhibit a clear LCST due to inherent solubility limitations: the C4 diamine‐based polyamide was excessively hydrophilic and readily water‐soluble, whereas the C6 analogue was entirely water‐insoluble due to its high hydrophobicity. Strikingly, the polyamide derived from cadaverine (C5 diamine) displayed intermediate behavior; it was water‐soluble at lower chain lengths, but its solubility decreased significantly with increasing molecular weight, becoming entirely water‐insoluble when the weight‐average molecular weight (*M*_w_) reached 44 200 g/mol. This solubility limit physically precluded the linear C5 system from reaching the requisite molecular weight for a distinct LCST. To overcome the solubility constraints of these linear polymers, we designed a star‐branched architecture using cadaverine. This approach involves coupling linear oligomers to a multifunctional core, achieving the high molecular weight required for a clear LCST while maintaining sufficient solubility. Crucially, unlike their linear counterparts, these star‐branched polymers exhibit significantly reduced chain entanglement and lower intrinsic viscosity even at high concentrations. Additionally, the dynamic coordination of pyrrolidones with water and metal ions allows for the retention of sufficient free water. This is driven by their structural character, where a single polar carbonyl serves as the sole coordinative group, efficiently projecting outward from the ring. Advantageously, the star‐branched architecture induces high chain mobility, decoupling the physical requirement of a leak‐free pseudosolid from the penalty of slow responsiveness. This strategy successfully addresses a long‐standing limitation of conventional responsive polymers.

Guided by this design principle, a series of fully bio‐based star‐polyamides was synthesized from IA and cadaverine. To ensure precise stoichiometric control and suppress potential side reactions from the vinyl group of IA, we employed a successful salt‐monomer formation methodology followed by thermal cyclization to form the pyrrolidone ring, yielding well‐defined amino acid monomers and oligomers (Scheme S1). Subsequently, target branched polymers with varying architectures—diamino (2 W), triamino (3 W), and tetraamino (4 W) branch centers were synthesized. The amide groups of the resultant branched polymers were confirmed around 1710 cm^−1^ (amide I) by FT‐IR (Figure S4). Number‐average molar mass (*M*_n_), weight‐average molar mass (*M*_w_), their distribution (Đ, *M*_w_/*M*_n_) (Figure S5), feeding ratio, and ζ‐potential are summarized in Table 1. The *M*_W_ and *M*_n_ of the branched polyamides ranged between 27900−31100 g/mol and 8600–9200 g/mol, respectively. *M*_w_/*M*_n_ ranged between 3.24 and 3.38 compared with oligomer 1.41, which was rational for the branched polymers. The typical ^1^H and ^13^C NMR spectra of branched polyamides are shown in Figure S6. They show the characteristic peaks of pyrrolidone at 2.53, 3.07, and 3.61 ppm. The proton signals of the branch center were located at 1.51 ppm for 2 W (Figure S7), 2.50 ppm for 3 W (Figure S8), and 3.26 ppm for 4 W (Figure S9). The *M*_n_ of arms can be calculated by the integral ratio of repeating units to branch center divided by core/arm ratio (with standard deviations now provided, e.g., 0.97% ± 0.21% for 4 W), as 7500 for 2 W,10 000 for 3 W, and 11 500 for 4 W. Notably, the absolute *M*_n_ values determined via ^1^H NMR end‐group analysis are in strong agreement with the theoretical design, confirming the effective participation of the multifunctional core and successful monomer conversion. While the NMR‐derived *M*_n_ closely correlates with the values estimated by Gel Permeation Chromatography (GPC), the GPC results are systematically slightly lower due to the intermolecular hydrogen bonding induced decreasing molecular volume detected by GPC. This can also be confirmed by the fact that increasing the number of arms elevates the local density of hydrogen‐bonding sites. This amplifies intramolecular hydrogen bonding, which compacts the polymer structure and reduces the apparent *M*_n_. Simultaneously, enhanced intermolecular hydrogen bonding induces transient aggregation, which drastically inflates the apparent *M*_w_ due to its high sensitivity to large species. Consequently, this simultaneous depression of *M*_n_ and inflation of *M*_w_ synergistically broadens the distribution. To accurately resolve the absolute molecular weight and overcome these complex solvent and interaction artifacts, Size Exclusion Chromatography‐Multi‐Angle Light Scattering (SEC‐MALS) analysis was performed in N, N‐dimethylformamide (DMF) (Figure S10). The true absolute *M*_w_ was found to be significantly lower than the apparent GPC estimates, ranging from 142 00 to 16 100 g/mol (Table 1). This discrepancy confirms that hexafluoroisopropanol (HFIP) in conventional GPC forces the branched arms into a highly extended conformation. Moreover, the GPC relies on linear poly(methyl methacrylate) (PMMA) as calibration standards. Because star‐branched polymers possess a significantly denser and more compact architecture than linear polymers, evaluating their hydrodynamic volume against linear PMMA standards inherently leads to an overestimation of their apparent molecular weight. In contrast, SEC‐MALS in DMF accurately captures the true dense, compact star‐branched topology without relying on linear references. To physically corroborate this star‐branched architecture, viscometric analysis revealed an exceptionally low intrinsic viscosity [η] of 1.83 ± 0.14 mL/g (Figure S11b) and distinct shear‐thinning behavior (Figure S11a), which are defining hallmarks of star polymers compared to linear analogs. Furthermore, the thermal properties of the polymers were evaluated via a thermal gravimetric analyzer (TGA), confirming excellent thermal stability with onset degradation temperatures (*T*_do_) remaining highly stable up to approximately 400°C (Figure S12a). Meanwhile, Differential Scanning Calorimetry (DSC) analysis revealed distinct glass transition temperatures (*T*_g_) of 51.2°C for 4 W, 72.1°C for 3 W, and 86.8°C for 2 W (Figure S12b). The trend of *T*_g_ increasing as the degree of branching decreases highlights the enhanced chain flexibility imparted by the highly branched architecture. Additionally, the DSC thermograms demonstrated that these star‐branched polymers exhibit similar melting points (*T*_m_). ^13^C NMR supported these structures (Figure S7–S9). As a result, the star‐branch structures of each polymer were well defined by the above confirmation.

**TABLE 1:** Molecular structure and colloidal properties of star‐polyamides ^[^ ^a^ ^].^

samples	core/arm ratio^b^	*M*_n__NMR_ (g/mol)^b^	*M*_n GPC_ (g/mol)^c^	*M*_w GPC_ (g/mol)^c^	*Đ* ^c^	*M*_w__SEC‐MALS_ (g/mol)^d^	ζ−potential (mV)^e^	*T_c_* ^f^
oligomer	N/A	N/A	4800	6750	1.41	6260	0.71±0.41	—
2W	2.67%±0.26%	8000	8600	27900	3.24	14200	4.76±0.24	30
3W	1.33%±0.22%	10000	8900	29600	3.32	15800	6.34±0.25	27
4W	0.97%±0.21%	11000	9200	31100	3.38	16100	9.45±0.14	25

^a^ The nomenclature is as follows: 2 W, 3 and 4 W refer to 2‐arm, 3‐arm and 4‐arm polymers.

^b^ Core/arm ratio and number‐average molar mass (*M*_n_ NMR) of arms were calculated by comparing the integrals of the typical group of branch center and the pyrrolidone group of arm.

^c^ Number‐average molar mass (*M*_n_ GPC), weight‐average molar mass (*M*_w_ GPC), and polymer dispersity index (Đ) were estimated by GPC using HFIP as the eluent.

^d^ Weight‐average molar mass (*M*_w_ SEC‐MALS) estimated by SEC‐MALS using DMF as the eluent.

^e^ ζ‐potential of polymer aqueous solution with a concentration of 2 wt.% at 25°C was determined by DLS analysis

^f^ *T*_c_ refers to the clouding temperature of the polymer aqueous solution with a concentration of 2 wt.% determined by UV–vis spectroscopy. *T*_c_ was undetectable between 0–90°C for the oligomer.

### 2.2 Thermoresponsive Behavior

The branched architecture of the synthesized star‐polyamides should serve as a powerful molecular dial to precisely control their thermoresponsive behavior, although the original polyamide oligomer did not show thermoresponsiveness. The thermoresponsive clouding phenomena of the star‐polyamides were systematically analyzed across a concentration gradient (5‐20 mg mL^−^^1^). The ζ‐potential, a key indicator of colloidal stability, increased systematically with branching density, from +4.76 mV for 2 W to +9.45 mV for 4 W (Table 1), which is not a very high value, while the trend can be regarded as evidence of star‐branching architectures. Crucially, the clouding temperature (*T*_c_, defined as 30% transmittance) was also directly tunable by the branching structure. As the branching degree increased from 2 to 4 W, the *T*_c_ decreased from 30°C to 25°C (2 wt.% in water) (Table 1). The *T*_c_ decrease is independent of almost constant *M*_n_ but dependent on *M*_w_, which means branched architecture is effective in controlling *T*_c_, although the positive charge of the arm end group does not seem effective. This can be attributed to the increased local density of polyamide arms to enhance relative hydrophobicity and inter‐arm interactions, which facilitate dehydration and phase separation at a lower thermal energy input. This demonstrates a powerful and predictable synthetic handle for fine‐tuning the thermoresponsive switching temperature of the material.

As exemplified by the 4 W polymer, aqueous solutions remain perfectly transparent at temperatures conducive to human comfort (<25°C) yet undergo an abrupt transition to a highly opaque state upon heating (Figure 1a). Heating above the *T*_c_ disrupts the hydration shell, triggering dehydration and leading to the aggregation of polymer chains into well‐defined, monodisperse coacervate droplets with a uniform diameter of 5.0 ± 0.3 µm, as visualized by optical microscopy (Figure 1b). Fundamentally, the uniform micro‐coacervates benefit from the unique stabilizing advantage of the charged star‐branched architecture. Unlike the classic coil‐to‐globule transition of linear PNIPAM that leads to unchecked macroscopic precipitation, these charged star‐polymers form well‐uniform droplets via a self‐arresting mechanism. As the droplets coalesce, the interfacial concentration of charged arm ends rapidly increases until electrostatic repulsion completely halts further growth. Consistent with this arrested growth, the coacervate‐size histograms monitored over ageing time (Figure S13) reveal a highly uniform size distribution that remains constant without shifting toward larger aggregates, further confirming the exceptional physical stability of the coacervate state. Below the *T*_c_, the polymers are fully solvated, and clouding behavior can be discussed due to entropy‐driven dehydration as *T*_c_ decreases with increasing concentration (Figure 1c). Branching density systematically governs *T*_c_, with values descending from 30°C (2 W) to 27°C (3 W) and 25°C (4 W) as shown in Figure 1d. This progression reflects enhanced intramolecular cooperativity and excluded volume effects in higher‐order architectures, which thermodynamically stabilize the dehydrated state. The transitions maintain exceptional fidelity through 30 heating‐cooling cycles (Figure 1e), demonstrating operational robustness for practical implementation.

**FIGURE 1:**
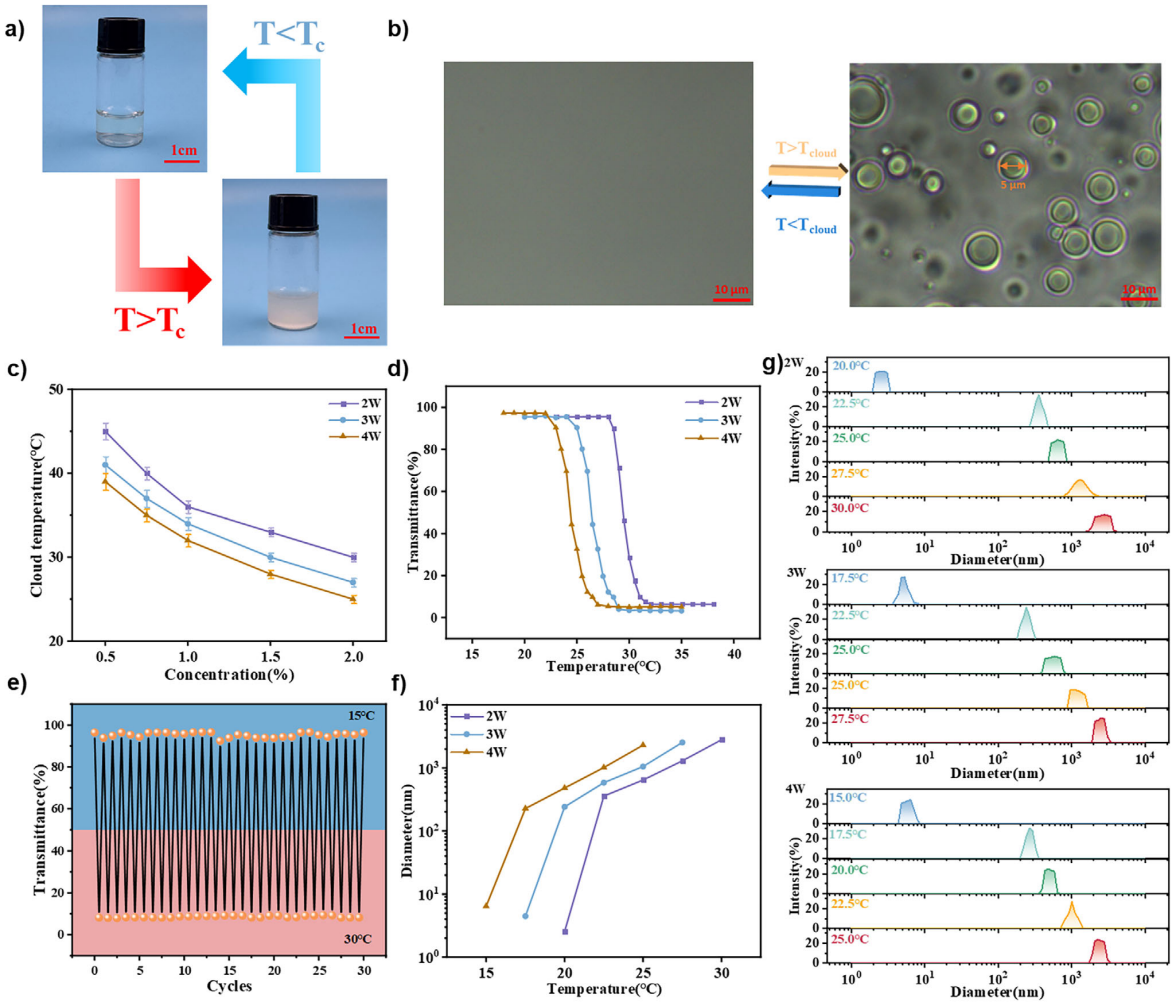
Thermoresponsive coacervation behavior of star‐polyamide aqueous solution. (a) Photographs of 4 W below and above *T*_c_; (b) Microscopic images of 4 W before and after *T*_c_; (c) Polymer concentration‐dependent *T*_c_ of 2, 3 and 4 W; (d) Temperature‐dependent transmittances of 2, 3, and 4 W; (e) 30‐cycle tests of thermoresponse for 4 W on heating to 30°C and cooling to15°C. (f) Diameters and (g) Diameter distributions of 2, 3 and 4 W at different temperatures.

Complementary Dynamic Light Scattering (DLS) analysis (Figure 1f,g) provides mechanistic insight into the phase transition, revealing a dramatic three‐order‐of‐magnitude size increase from ∼3–6 nm to ∼2.5 µm upon heating. This growth, synchronized with optical turbidity development, signifies a coherent microphase separation driven by entropic polymer‐solvent incompatibility. The perfect concordance between turbidimetry and light scattering inflection points validates this unified transition model. This programmable optical switching is the cornerstone of the material's smart window functionality. The transparent state allows high visible light transmittance. In contrast, the opaque state efficiently scatters both visible and near‐infrared radiation, thereby providing passive, self‐regulating control over solar heat gain to improve building energy efficiency significantly.

Beyond establishing the topological characteristics of the star‐branch polyamides through their arm number and degree of branching, it is equally important to consider the macroscopic dimensions of the polymer chains. We further demonstrate that molecular weight serves as an independent, powerful parameter for programming LCST behavior. 4 W polyamides across a molecular weight range of 11 400–31 100 g/mol (Table S1 and Figure S14) maintain sharp optical transitions while exhibiting *T*_c_ depression from 51°C to 25°C with increasing molecular weight. This molecular weight dependence follows classical LCST thermodynamics based on Flory‐Huggins theory, where larger chains experience greater entropic gains upon dehydration and collapse, thermodynamically destabilizing the solvated state. Consequently, less thermal energy is required to overcome the barrier for phase separation, driving the *T*_c_ to lower temperatures as the chain length increases. As shown in Figure S15a, the reversible optical changes from transparent to turbid were observed while the temperatures were lower or higher than *T*_c_. Concentration‐dependent studies (Figure S15b) confirm this trend across all molecular weights, while transmittance profiles (Figure S15c) reveal maintained transition cooperativity despite varying *T*_c_. DLS (Figures S15d and S16) quantifies the structural basis of these transitions, showing consistent size increases from nanoscopic solvated chains (∼3–6 nm) to microphase‐separated domains (∼2.5 µm) across the molecular weight series. These findings establish multiple orthogonal parameters, including branching architecture, molecular weight, and concentration for precise tuning of thermoresponsive behavior.

### 2.3 Molecular Mechanism

To elucidate the molecular‐scale events underlying the clouding transition, we employed ^1^H NMR spectroscopy in an aqueous environment at variable temperature (Figure 2a). Quantitative analysis of normalized signal intensities, *I*/*I*_0_, where *I* and *I*_0_ refer to the intensities at the measurement temperature and 25°C, respectively, to clarify a cooperative dehydration mechanism (Figure 2b). In the ^1^H NMR spectra, it is noted well‐resolved characteristic signals (unit: ppm) of the representative structure of hydrophilic pyrrolidone are noted, marked as **c** in a chemical shift range of 3.43–3.71 ppm, and as **b** in a chemical shift range of 2.53–2.71 ppm (CH_2_, pyrrolidone ring), and of hydrophobic alkylene marked as **a** in a chemical shift range of 1.21–1.70 ppm. The integral ratio of *I*/*I*_0_ normalized by the internal standard 4,4‐dimethyl‐4‐silapentane‐1‐sulfonic acid (DSS) integral value (Figures S17–S19), for signals **a**, **b**, and **c** drops drastically around 32°C. As the temperature surpasses the cloud point identified by bulk measurements, all proton signals undergo synchronous and precipitous attenuation and decrease to approximately 60% of their original intensity by 35°C, perfectly corroborating the dynamic macro‐droplets observed under optical microscopy. This uniform signal collapse demonstrates concerted dehydration of both hydrophobic and hydrophilic segments, culminating in the formation of phase‐separated aggregates that cause signal decay due to restricted molecular motion. Notably, this sharply contrasts with conventional linear polymers like PNIPAM, whose signals typically vanish (<30%) due to bulk solid aggregates. The substantial residual signal in our system indicates that these phase‐separated domains remain as dynamic, liquid‐like coacervates. By retaining significant molecular mobility and a substantial fraction of free water within the collapsed state, the star‐polymers ensure rapid, reversible thermoresponsiveness while effectively circumventing kinetic trapping. Moreover, this dynamic hydration state is quantitatively supported by Low‐Field Nuclear Magnetic Resonance (LF‐NMR) analysis (Figure S20). Specifically, the transverse relaxation time (*T*_2_) distribution curve resolves two distinct water populations: a minor peak at shorter relaxation times (∼100 ms) corresponding to the tightly bound water (quantified as q = 59.45 mg/g for the 4W‐2 M system), and a dominant peak at longer relaxation times, which explicitly confirms the presence of abundant, highly mobile residual free water within the coacervate microdomains. The perfect temporal correlation between NMR signal decrease and hydrodynamic size increase establishes an unequivocal chain collapse mechanism, directly connecting molecular‐scale dehydration to macroscopic property changes. Furthermore, temperature‐dependent Fourier Transform Infrared spectroscopy (FTIR) (Figure S21) and two‐dimensional correlation spectroscopy (2Dcos) (Figure S22) were employed to probe the change in hydrogen bonding during heating. As illustrated in Figure 2c, the 1D FTIR spectra in the 1800–1500 cm^−1^ region exhibit distinct variations as the temperature increases from 25°C to 45°C. The prominent absorption bands at approximately 1630 and 1560 cm^−1^ are assigned to the Amide I band (C = O stretching vibration) and the Amide II band (N─H bending and C─N stretching vibrations), respectively. With elevating temperature, the blue shift of the C = O band and red shift of N─H band provide compelling molecular‐level evidence for the phase transition pathway of the thermoresponsive star‐polyamide. In the fully hydrated state below LCST (25°C), the polyamide chains are extended, and the Amide I band is completely dominated by a major peak (79.4%) centered at approximately 1630 cm^−1^, assigned to the highly hydrated C = O groups forming strong hydrogen bonds with water (polymer‐ H_2_O hydrogen bonding). The minor peak (12.6%) at 1655 cm^−1^ suggests the presence of a small fraction of pre‐existing intermolecular polymer‐polymer C = O interactions. Similarly, in the Amide II region, the spectrum is primarily characterized by the hydrated N─H bending vibration (8.0%) at 1560 cm^−1^ (Figure 2d). As the temperature rises and the thermal energy overcomes the enthalpy of hydration, the distribution of these microstates undergoes a dramatic inversion, clearly illustrating the thermally induced dehydration and subsequent physical crosslinking. The area of the hydrated C = O peak significantly decreases (18.1%), while the intermolecular hydrogen‐bonded C = O peak experiences massive growth (61.4%), shifting the overall band envelope to higher wavenumbers (a macroscopic blue shift). Concurrently, a new highly pronounced peak emerges at 1545 cm^−1^, corresponding to the formation of dense, intermolecular hydrogen‐bonded N─H groups (14.7%), at the expense of the hydrated N─H peak (5.8%). This quantitative redistribution of peak areas provides direct evidence that the polymer chains expel their hydration shells to assemble into polymer‐rich coacervate droplets bound by newly formed intermolecular (C = O···H─N) networks. While the 1D deconvolution confirms the transition from a water‐bound to a polymer‐bound state, generalized 2Dcos was employed to determine the precise sequence of these molecular events. In the synchronous 2D correlation spectrum (Figure 2e), prominent positive auto‐peaks are observed on the diagonal at 1630 and 1560 cm^−^^1^, confirming that both functional groups are highly sensitive to thermal stimuli. The presence of a strong positive cross‐peak at (1630, 1560) demonstrates that the dynamic variations of the C = O and N─H groups proceed in the same direction—specifically, simultaneous structural evolution as the temperature traverses the LCST phase boundary. The asynchronous spectrum provides the temporal order of structural variations during the heating process (Figure 2f). Based on Noda's rules [43, 44] and the cross‐peak signs in the asynchronous map, the specific sequence of group responses is unambiguously determined to be: Hydrated C = O (1630 cm^−1^) → Intermolecular C = O (1655 cm^−1^) → Hydrated N─H (1565 cm^−1^) → Intermolecular N─H (1545 cm^−1^). This precise sequence reveals a highly coordinated, carbonyl‐led dehydration mechanism [45, 46] during coacervation. This carbonyl‐led dehydration sequence is intrinsically driven by the constrained topology of the star‐branched architecture with extremely high local concentration of pyrrolidone rings. The outward‐projecting C = O of the pyrrolidone ring acts as the highly accessible primary hydration site, whereas the N─H groups are sterically shielded by adjacent bulky rings and aliphatic chains. Consequently, heating first disrupts the exposed C = O hydration shell to trigger initial chain association, which subsequently forces the desolvation of the protected N─H domains. Immediately following this local desolvation, these newly dehydrated N─H groups lock into the assembling polymer network, forming stable intermolecular N─H hydrogen bonds that finalize the dense internal structure of the coacervate droplets. This continuous sequential breakdown confirms that the macroscopic phase transition is not an instantaneous collapse, but rather a kinetically choreographed assembly initiated specifically by the desolvation of the carbonyl groups.

**FIGURE 2:**
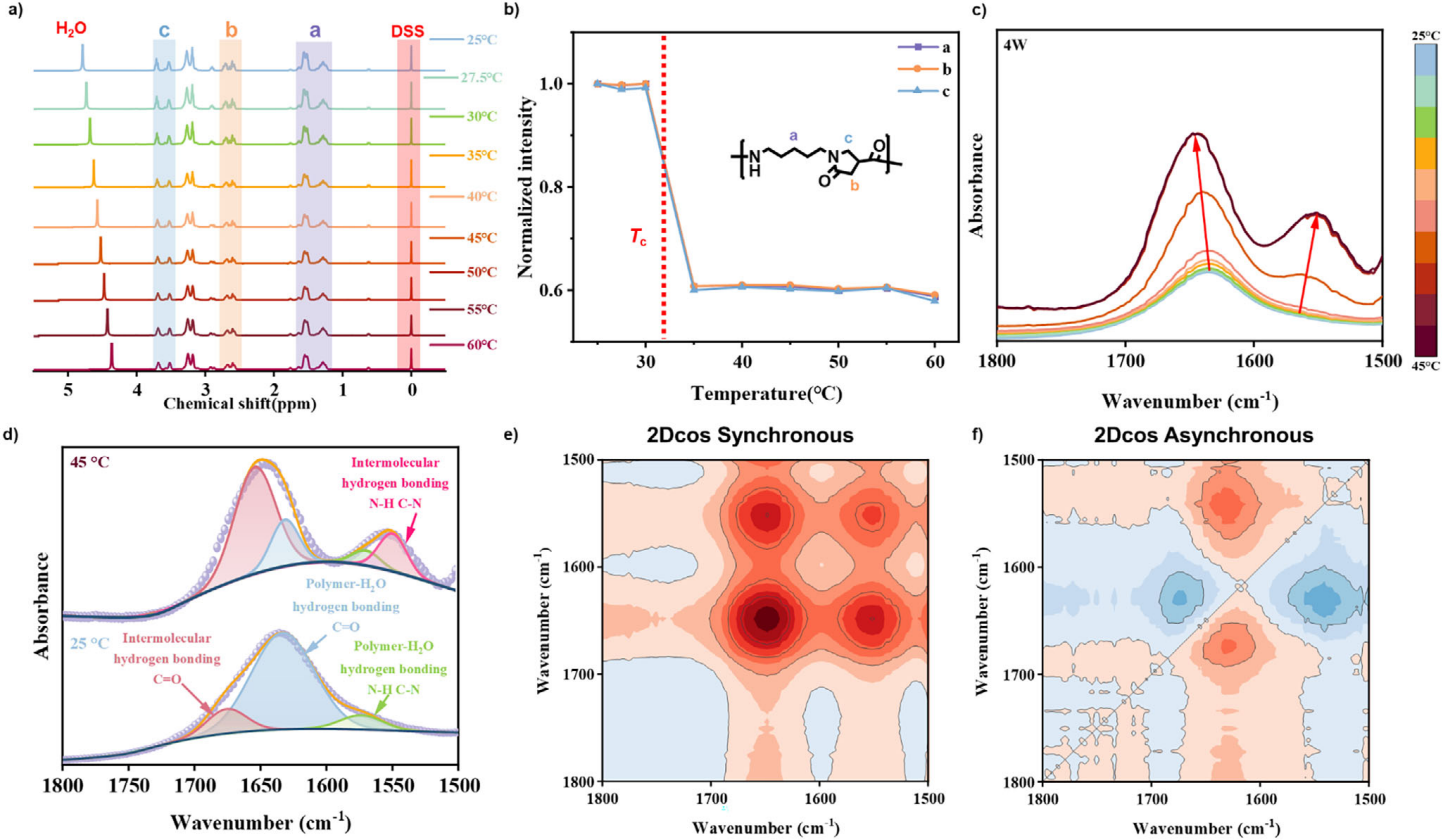
^1^H NMR analyses of star polymer (4 W) in D_2_O at various temperatures from 15°C to 60°C. (a) ^1^H NMR spectra. (b) Normalized intensity change showing a drop around 32°C, corresponding with *T*_c_. (c, d) Temperature‐dependent FTIR spectra of the 4 W solution upon heating from 25°C to 45°C. (e, f) 2Dcos synchronous and asynchronous spectra.

### 2.4 Ion‐Dependent Thermoresponsive Behaviors

#### 2.4.1 Salt Effects

We report a remarkable dichotomous ion‐specific response in star‐polyamides. It is well established that anions such as Cl^−^ exhibit a pronounced salting‐out effect by disrupting the hydrogen bonding between polymer chains and water molecules [47]. Consequently, in the NaCl system, the synergistic combination of Na^+^ and Cl^−^ induces classical salting‐out behavior with *T*_c_ decreasing from 30°C to 8°C with increasing ionic strength, as shown in Figure S23a. On the other hand, despite the presence of the salting‐out Cl^−^, the anion Ca^2+^ produces a potent salting‐in effect, elevating *T*_c_ from 25°C to 66°C (Figure S23b). Figure 3a shows the *T*_c_ plots under various ionic strengths of both NaCl and CaCl_2_. The opposite salt addition effects are very clear, and notably, the present star‐branched polyamides have never precipitated under such a high concentration of CaCl_2_. Because both systems share the same Cl^−^ anion, this striking contrast effectively isolates the decisive role of the cations.

**FIGURE 3:**
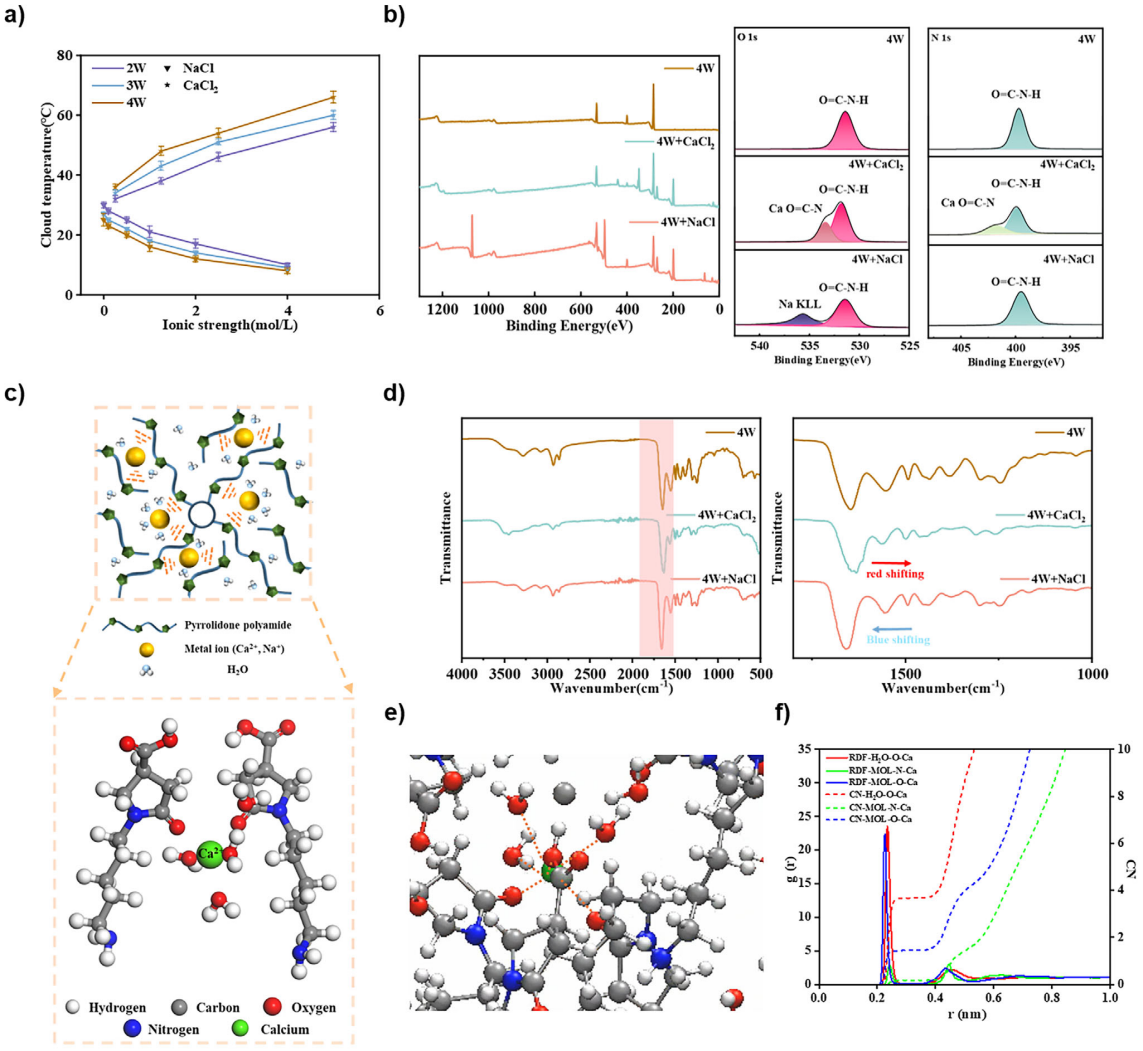
Thermoresponsive behavior of 4 W aqueous solution under NaCl and CaCl_2_. (a) *T*_c_ under different concentrations of NaCl and CaCl_2_ (b) XPS survey, O 1s peaks, and N 1s peaks spectra of 4 W, 4W+CaCl_2,_ and 4W+NaCl. (c) Graphic and 3D structural coordination diagram of pyrrolidone polyamide models with Ca and H_2_O by DFT calculation. (d) FTIR spectra of 4 W, 4W+CaCl_2,_ and 4W+NaCl. (e) Molecular dynamics diagram of pyrrolidone polyamide models with Ca and H_2_O in 20 ns. (f) RDF and CN of Ca with H_2_O and pyrrolidone molecules.

Ca^2+^ produces a pronounced branch‐dependent enhancement, where increasing arm number, namely pyrrolidone content, amplifies the *T*_c_ elevation (Figure S23e). Hydrodynamic diameter analyses (Figure S23c,d) of three branched polymers in different ion solutions were measured by DLS from 3 to 71°C, which also confirms the critical temperature, corresponding to *T*_c_. Additionally, two different trends of salt effect were absolutely meaningful to discover the different mechanisms of the two kinds of ions. For NaCl, the competitive hydration with H_2_O driven by both Na^+^ and Cl^−^ breaks the hydration layer on the polymer surface, leading to lower solubility and cloud temperature. Besides, this kind of effect in different branched polymers, with reference to the different arm numbers, the attenuation is almost the same. On the other hand, the mechanism of the opposite effects of CaCl_2_ was not simply explained. Then, under microscopic investigation (Supporting Video S1), the self‐coacervated polymer droplets exhibited a clear collapse upon the attack of calcium ions, providing a visual tentative reason for the influence of thermo‐performance and ionic coordination. In a striking anomaly to the Hofmeister series, Ca^2+^ induces a profound salting‐in effect due to its specific ionic radius and coordination geometry. It completely overrides the inherent salting‐out tendency of the surrounding Cl^−^ anions by uniquely co‐coordinating. It uniquely co‐coordinates the pyrrolidone carbonyl and water, acting as a dynamic crosslinker that recruits water and exceptionally stabilizes the polymer's hydration shell. Additionally, DLS analysis and microscopic images (Figure S24) reveal that even under Ca^2+^, the solution effectively suppresses aggregate growth at high temperature as uniformly, controlled‐size droplets. We further confirm this phenomenon by significantly enhancing aqueous solubility (>50 wt.%). However, for practical applications, using highly condensed polymer solutions generally exhibiting extremely low *T*_c_, precisely modulating the phase transition to an appropriate ambient *T*_c_ is critical. To achieve this, we employed a mixed‐ion strategy. To overcome the severe kinetic trapping caused by extreme viscosity at such concentrations, we determined clear, reversible *T*_c_ transitions by employing prolonged isothermal equilibration at each temperature step. Competitive ion experiments (Figure S23f) demonstrate that introducing Na^+^ partially disrupts the Ca^2+^‐pyrrolidone coordination, acting as an ionic counter‐program, but it still keeps the Ca^2+^ solubility enhancement. Crucially, in an equimolar (1:1) mixture of Na^+^ and Ca^2+^, this antagonistic interplay effectively tailors the phase transition; the resulting *T*_c_ is modulated to a desired working range while remaining 21°C higher than that of a pure NaCl solution. This successful measurement highlights the exceptional structural stability and retained chain mobility of the Ca^2+^‐star‐polymer system. This confirms the exceptional stability of the Ca^2+^ complexes whose function is not hindered by Na^+^, while proving that strategically mixing specific ions allows for the precise modulation of *T*_c_ at high concentrations, opening new pathways for designing advanced, highly concentrated smart materials.

#### 2.4.2 Molecular Coordination

To elucidate the fundamental mechanisms underlying the contrasting ion effects, we employed a combined computational and spectroscopic approach. The results of molecular simulation are shown in Figure 3c and Figures S25 and S26. DFT simulations reveal that both Na^+^ and Ca^2+^ form octahedral coordination spheres, yet with critically different binding preferences (Table 2). A comparison of the calculated coordination energy values reveals that the calcium ion interacts more strongly with water molecules than the sodium ion. A particularly noteworthy point is that the most stable configuration occurs with the combination of a calcium ion, four water molecules, and two polymer models. This suggests that even though a Ca^2+^ ion typically coordinates with six water molecules in the bulk of the solution, approaching a polymer chain causes an exchange of the water molecule with the polymer chain. This specific co‐coordination state represents a delicate thermodynamic equilibrium, where the coordinating force of the pyrrolidone carbonyl oxygen to Ca^2+^ effectively balances the coordinating force of water. Exchanging a portion of the Ca^2+^ hydration shell with the polymer minimizes the system's overall free energy, allowing the Ca^2+^ ion to act as a dynamic crosslinker while retaining sufficient water to preserve the hydration sphere. Furthermore, based on this most stable structure, there is a possibility for the number of coordinating polymer chains to be variable between one and three **(entries 5–7 in** Table 2). Hence, the affinity of pyrrolidone toward Ca^2+^ falls below the threshold required to completely dismantle the Ca^2+^ hydration sphere. Consequently, branched architectures with higher arm numbers, that is, a larger amount of pyrrolidone, display greater solubility under Ca^2+^ coordination, resulting in the largest *T*_c_ increase. These phenomena are further substantiated by ζ‐potential measurements at 50°C (Table S2). It was observed that a higher number of arms in the star‐polyamides facilitated the coordination of a greater quantity of Ca^2+^ ions, which in turn resulted in a more positive ζ‐potential value. To rule out the possibility that these elevated positive charges originate merely from inherent amine protonation, pH‐dependent ζ‐potential measurements were also conducted (Table S3). Although the polyamides exhibited expected highly positive potentials under acidic conditions due to protonation (+16.48 mV for 4 W at pH 3), the highly positive ζ‐potential induced by the addition of 0.5 M CaCl_2_ (+18.39 mV) vastly exceeds the native neutral state of the 4 W polymer (+9.45 mV at pH 7). This confirms that the anomalous positive charge is predominantly driven by the specific interfacial coordination of Ca^2+^ ions onto the polymer chains, rather than simple protonation effects. In contrast, Na^+^ with its weaker coordination strength fails to induce this anomalous trend. Instead, it competes with the polymer for hydration, destabilizes the aqueous microenvironment, and monotonically reduces the *T*_c_. As summarized in Table S2, the ζ‐potential of the 4 W polyamide in the presence of Na^+^ exhibited a significant decrease. This reduction is attributed to the poor coordination affinity between the pyrrolidone rings and Na^+^. Consequently, instead of being further cationized, the intrinsically positively charged 4 W chains preferentially associate with counter‐ions Cl^−^, leading to a noticeable decline in the absolute value of the zζ‐potential. On the other hand, the coordination energy differences (Δ*E*) between the polymer–ion–water ternary systems and the binary polymer–water systems provide a quantitative energetic rationale for the observed behavior. The Δ*E* for the Na^+^ system was determined to be −110.57 kcal/mol (calculated by subtracting **Entry 0 from Entry 3 in** Table 2). In contrast, the stable Ca^2+^ coordination system exhibited a significantly more negative value of −217.22 kcal/mol, derived from Entries 0 and 6. This substantial disparity in binding energy originates from the lower charge density of the monovalent Na^+^ ion, which results in a significantly weaker electrostatic interaction energy compared to the divalent Ca^2+^. Consequently, Na^+^ lacks the sufficient thermodynamic driving force required to break its strong existing coordination bonds with water molecules to form a new complex with the pyrrolidone ring. This explains why Ca^2+^ effectively stabilizes the coordination complex and elevates the *T*_c_, while Na^+^, despite having a similar coordination geometry, possesses insufficient binding affinity to significantly modulate the phase transition. To better understand the molecular coordination and conformational dynamics of the polymers and Ca^2+^, molecular dynamics (MD) simulations were performed to investigate the interaction between the 4w polymer and CaCl_2_ in aqueous solution. As illustrated in Figure 3e and Figures S27 and S28, and Supporting Video S2, calcium ions spontaneously associate with the carbonyl groups on the pyrrolidone rings, forming a stable coordination structure. Radial distribution function (RDF) analysis (Figure 3f) revealed coordination numbers (CN) of 3.6 for Ca‐H_2_O and 1.4 for Ca‐4 W (Table S4). These values are slightly lower than theoretical ideals, attributed to steric hindrance within the bulky macromolecular system and competitive hydrogen bonding between the polymer and water. The results still indicated that the 4 W polymer and water molecules form a stable octahedral coordination structure centered on the calcium ion, with a coordination number of six, which was consistent with the DFT calculation results.

**TABLE 2:** Coordination energy between various molecule models of metal ion, H_2_O, and polymer minimum units (M).

entry	Molecular model	Coordination energy (kcal/mol)
0	2M+4 H_2_O	−39.49
1	Na+6 H_2_O	−106.67
2	1M+Na+5 H_2_O	−111.28
3	2M+Na+4 H_2_O	−150.06
4	Ca+6 H_2_O	−250.47
5	1M+Ca+5 H_2_O	−256.69
6	2M+Ca+4 H_2_O	−282.74
7	3M+Ca+3 H_2_O	−256.71

Furthermore, x‐ray Photoelectron Spectroscopy (XPS) analysis provides direct evidence of distinct coordination chemistries (Figure 3b). In the O 1s spectrum, Ca^2+^ coordination induces a 0.4 eV shift to higher binding energy for carbonyl oxygen (531.8 eV) and generates a unique coordination peak at 533.4 eV, which explicitly indicates the donation of electron density from the carbonyl oxygen lone pairs to the vacant orbitals of Ca^2+^. Concurrently, the N 1s spectrum reveals a new nitrogen environment at 401.8 eV, confirming that this primary charge donation to Ca^2+^ triggers a pronounced redistribution of electron density throughout the entire pyrrolidone ring, thereby pulling electron density away from the adjacent nitrogen atom. On the other hand, no such peak is observed for the 4W‐Na system. Instead, a Na‐specific Auger peak (Na KLL) appears at 535.5 eV for 4W‐Na. In the N 1s region, the 4W‐Ca also displays a shifting signal at 401.8 eV induced by Ca^2+^ coordination, which corresponds to the nitrogen atom neighboring the pyrrolidone C = O group. FTIR spectroscopy (Figure 3d) further corroborates these distinct interaction mechanisms. Ca^2+^ coordination produces a 15 cm^−1^ red shift from 1645 to 1630 cm^−1^ in the carbonyl stretching frequency. This perfectly links with the XPS findings: the withdrawal of electrons from the carbonyl oxygen toward Ca^2+^ weakens the C = O double bond, lowering its force constant and directly causing the observed macroscopic red shift. Conversely, Na^+^ causes a blue shift from 1645 to 1660 cm^−1^, indicating disrupted hydration and reduced electron density at the carbonyl group without charge‐transfer coordination. To provide direct macroscopic evidence for the retained hydration shell of the coordinated Ca^2+^ ions, LF‐NMR relaxometry was employed (Figure S29). The *T*_2_ distributions explicitly reveal and quantify the strongly bound water associated with the polymer chains in the calcium‐coordinated systems, demonstrating a substantial bound water capacity (q = 3.79 mg/g for the 4W–0.1 M Ca system and q = 2.70 mg/g for the 4W–2.0 M Ca system). This directly proves that upon coordinating with the polymer, Ca^2+^ ions successfully retain their distinct hydration shells. This comprehensive analysis resolves the paradoxical ion‐specific behavior: Ca^2+^ forms structured coordination complexes that establish reinforced hydration layers through charge‐assisted hydrogen bonding, thereby elevating T_c_. The stability of these complexes is maximized in highly branched architectures, explaining the amplified salting‐in effect with increasing arm number. In contrast, Na^+^ follows conventional Hofmeister behavior through competitive hydration and charge screening effects. This fundamental understanding of ion‐specific coordination mechanisms provides a new design principle for advanced stimuli‐responsive materials, enabling precise control over thermal transitions through selective ion incorporation.

### 2.5 Star‐Polyamides‐Based Smart Windows

A critical challenge for thermoresponsive smart windows lies in maintaining stable phase separation under operational conditions while withstanding complex environments. We evaluate star‐branched 4 W against conventional thermoresponsive polymers (PNIPAM and HPC) under ionic conditions (Figure S30). The 4 W solution exhibits enhanced stability compared to PNIPAM and HPC; while the latter phase‐separates within 20–40 min in saline, 4 W persists for 2h, presumably owing to its branched architecture with charged ends. This stability is even more pronounced in Ca^2+^ solutions, where 4 W maintains a homogeneous dispersion for unprecedented durations exceeding 8 h (Figure S31). This remarkable stabilization stems from the dynamic coordination between Ca^2+^ and pyrrolidone rings, forming a robust network that resists gravitational settling. As previously suggested by the MD and DFT, sodium ions seem to theoretically coordinate with the pyrrolidone rings; however, this interaction is much weaker compared to Ca^2+^. Thus, while Na^+^ predominantly induces competitive dehydration (salting‐out), the exceptional structural stability of the 4 W architecture delays macroscopic precipitation much longer than in conventional linear polymers.

Furthermore, to overcome the ubiquitous liquid leakage and environmental evaporation challenges that fatally plague typical aqueous smart windows (liquid solutions or solid hydrogels), we leveraged this ion‐coordination strategy to engineer a robust pseudosolid state. As illustrated in the comprehensive schematic (Figure S34a), this strategy fundamentally relies on distinct ion‐coordination mechanisms: in contrast to the weak coordination of Na ions (conventional salting‐out), Ca ions strongly coordinate with the branched polyamide to form a new hydration layer (anomalous salting‐in). This strong coordination enables the polymer to achieve a homogeneous soluble state even at an extreme concentration (>50wt.%). During the thermo‐responsive phase transition upon heating (T > *T*_c_), the polymer‐H_2_O hydrogen bonds dissociate, and intermolecular hydrogen bonding becomes dominant, driving the reversible aggregation of the polymer chains into particles. By utilizing a binary salt mixture (2 M CaCl_2_ and 0.2 M NaCl), we finely counter‐programmed the cloud point to a practical 35.1±0.7°C while formulating an ultra‐high‐concentration (50 wt.%) star‐polyamide system (Scheme 1 and Figure S32). As depicted in Figure S33 and Supporting Video S3, this extreme concentration fundamentally transforms the physical state into a pseudosolid material. To quantitatively validate this pseudosolid behavior, dynamic rheology measurements were performed. As shown in Figure S34b, the storage modulus (*G*′) consistently exceeds the loss modulus (*G*″) across a temperature range of 35°C to 50°C, confirming a dominant elastic, solid‐like network. Moreover, the material exhibits a distinct yield point at approximately 10 Pa, demonstrating that a threshold force is required to initiate flow, fundamentally characterizing it as a yield‐stress fluid (Figure S34c). This structural stability is further corroborated by quantitative creep tests (Figure S34d), where the material demonstrates an initial elastic response that quickly plateaus at a constant maximum shear strain of ∼13–14% over a prolonged period (>1100 s), showing no continuous deformation under constant stress. These rheological properties collectively impart an ultra‐high viscosity that effectively arrests macroscopic flow, thereby creating a highly stable and non‐leaking pseudosolid architecture. Crucially, owing to the unique star‐branched topology, this pseudosolid state perfectly balances macroscopic structural integrity with microscopic mobility, completely eliminating the risks of fluid leakage and rapid desiccation while preserving the stimuli‐responsiveness that conventional linear polymers inevitably lose at such extreme solid contents.

Beyond exceptional thermoresponsive stability, we engineer dual electro‐thermal responsiveness by integrating platinum electrodes into a 4 W window containing 0.5 M CaCl_2_ (Figure 4a). Applying 3 V potential induced reversible, spatially controlled transparency switching: calcium migration to the cathode‐maintained transparency, while its depletion at the anode triggered localized turbidity. Upon applying a 3 V current, the anode side gradually becomes cloudy (Supporting Video S4). This electro‐responsive behavior, also confirmed by polarity reversal experiments and coacervate particle tracking, enables active smart window control independent of ambient temperature. Further, we verify this unique phenomenon by analyzing the visible light transmittance versus time curves of the 4 W polymer under different voltages (Figure 4b). After voltage application, the 4 W polymer solution completes its response and reaches a steady state within 20–30 s. Specifically, a 10 V application rapidly made the window fully turbid in around 6 s. This rapid response speed is fundamentally determined by the electrophoretic driving force (drift speed) of the Ca^2+^ ions under the applied electric field, followed by the near‐instantaneous dissociation and reconstruction of the dynamic coordination network between the migrating Ca^2+^ and the polymer chains. It is imperative to emphasize that this electro‐responsive mechanism is uniquely enabled by our ion coordination system. In stark contrast, conventional thermoresponsive polymers like PNIPAM or HPC cannot exist as stable solutions under such high ionic concentrations; they suffer from immediate, irreversible salting‐out and phase separation, rendering any active electrophoretic control impossible. Importantly, despite the application of continuous voltage, the system demonstrated excellent electrochemical stability. To quantitatively validate this cycling durability, continuous in situ UV–vis monitoring demonstrated highly consistent and reversible optical profiles over 30 consecutive electro‐responsive cycles, maintaining robust transmittance amplitude without noticeable decay (Figure S35). Although equipment constraints limited continuous in situ monitoring to 30 cycles, UV–vis testing after 100 rapid cycles confirmed the material fully retains its excellent optical switching reversibility without amplitude decay (Figure S36b). Furthermore, post‐cycling ^1^H NMR analysis of the 4 W polymer after 100 complete cycles revealed spectra nearly identical to the pristine material (Figure S36a). All characteristic proton peaks remained perfectly intact without shifting, loss of integrals, or the appearance of new byproduct peaks, definitively confirming that no structural material degradation or disruptive electrolysis occurs during extended operation. No significant material degradation or disruptive electrolysis was observed during the electro‐responsive cycles. Together with the observed movement of coacervation particles under microscopy (Supporting Videos S5 and S6), during the migration of calcium ions under the electric field, the dynamic coordination network forms between the pyrrolidone rings within the coacervated droplets of the 4 W polymer, and the calcium ions also migrate accordingly. As a result, the coacervated particles are driven to move directionally by the electric field, which aligns with our predicted mechanism of electrical responsiveness. This experiment effectively verifies the electrical controllability of the electro‐thermally responsive smart window.

**FIGURE 4:**
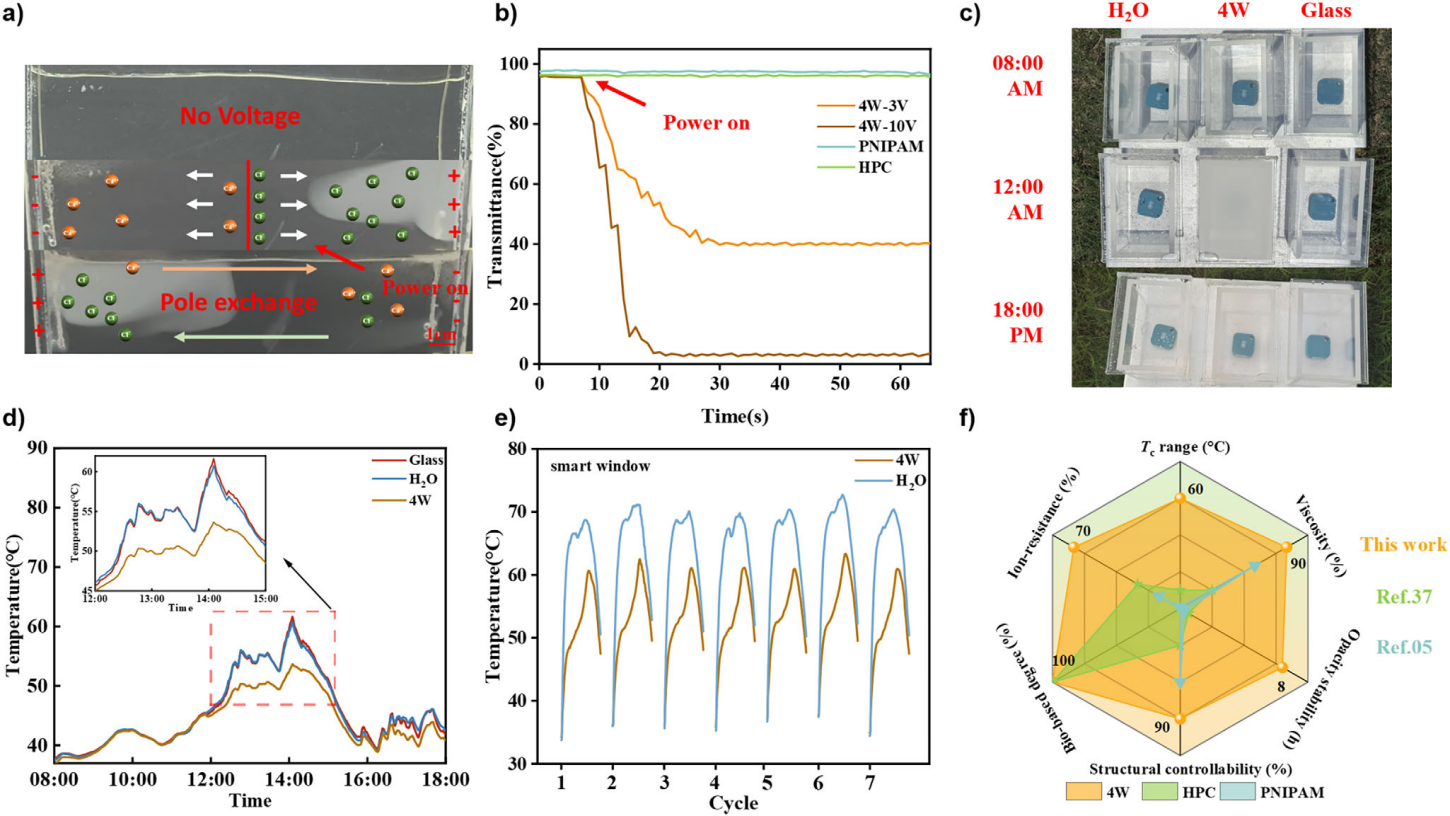
Star‐branched polyamide application for dual‐stimuli responsive smart windows. (a) Electrically‐functional smart window of 4 W polymer under 3 V. (b) Time‐dependent transmittances of 4 W, PNIPAM and HPC aqueous solution under the 3 and 10 V. (c) Photos of the outdoor simulation test setup with a test compartment, a 4 W smart window, an H_2_O window, and control with a normal window at different times, placed at the university campus (Wuxi, China) in summer. (d) Real‐time temperature evolution curves of the test. (d) Temperature changes of the room with a water‐including cell lid agreed with those with a glass lid, while the lid including 4 W solution was effective in restricting the temperature increase. (e) Cyclic real‐time temperature evolution curves of 4 W and H_2_O smart windows. (f) A hexagon radar plot showing the excellent comprehensive performance of 4 W.

Real‐world performance is validated through summer field tests in Wuxi, China (Figure 4c). The 4 W smart window autonomously modulates solar gain, maintaining transparency during cool morning hours, switching to an energy‐blocking opaque state at noon, and fully recovering its high transparency in the evening (18:00) as ambient temperatures decrease. It should be noted that the slight visual blurriness observed on the exterior enclosures in the 18:00 photographs is an artifact caused by light scattering from residual rainwater droplets (Figure S37) following a brief afternoon shower, whereas the internal 4 W polymer successfully achieved complete, reversible transparency. Most impressively, it achieves an 8°C temperature reduction under peak irradiation (53.4°C vs 61.5°C in control) and maintains an average 5.1°C cooling advantage throughout daytime operation (Figure 4d). Moreover, during the week‐long cycling tests (Figure 4e), the 4 W polymer‐based smart window demonstrates notable stability and maintains rapid switching performance across both high and low temperatures, enabling efficient and reliable temperature regulation. It is important to clarify that the apparent difference in baseline and peak temperatures between Figure 4d,e (peak temperatures of ∼53.4°C and ∼60°C for the 4 W sample) originates from the distinct weather conditions on the respective testing dates. While Figure 4d corresponds to a rainy day, Figure 4e records performance during typical clear and highly irradiated hot summer days, reflecting a significantly higher ambient solar load. The excellent comprehensive performance of 4 W polymer is summarized and compared with other thermoresponsive polymers (Figure 4f). Here, the *T*c range is defined as the controllable cloud temperature range. The ion‐resistance is defined by the ability to retain performance in ionic environments. Viscosity reflects the solution consistency and flow resistance, respectively.

### 2.6 Life Cycle Assessment of Star‐Branched Polyamides

To quantify the system‐level climate and sustainability benefits of the star‐branched bio‐based polyamide (Bio‐PA) platform, we conducted an AI‐assisted cradle‐to‐gate Life Cycle Assessment (LCA) (Carbon AI, carbonai.carbonstop.com) in compliance with ISO 14067:2018, the GHG Protocol, and the ISO 14064 series of standards [48, 49]. We benchmarked the net carbon footprint of the industrially scaled Bio‐PA system against conventional petroleum‐derived thermochromic polymers (PNIPAM) and semi‐synthetic controls (HPC). To ensure a robust and holistic evaluation, the LCA system boundaries were comprehensively expanded beyond raw material acquisition and process utility energy to encompass biogenic carbon sequestration, catalysts and additives, solvent makeup and losses, and waste/wastewater treatment streams. This rigorous assessment aligns with the sustainable material sourcing and production principles required for SDG 12. The LCA results reveal distinct cradle‐to‐gate carbon footprints for the evaluated monomeric precursors and polymer systems (Figure 5a and Figures S38–S42), with the industrially scaled bio‐based polyamide pathway delivering profound system‐level climate benefits. Traditional thermochromic systems rely heavily on petroleum‐derived monomers; for instance, the synthesis of N‐isopropylacrylamide (NIPAM) and Propylene Oxide (PO) strictly adds to the carbon burden, yielding zero biogenic sequestration and resulting in the highest net carbon footprints (+3.05 and +2.30 kg CO_2_e/kg, respectively). Consequently, the conventional PNIPAM control exhibits a highly positive net life cycle emission (+7.26 kg CO_2_e/kg), driven by the carbon‐intensive raw material acquisition phase (+4.90 kg CO_2_e/kg), significant solvent makeup burdens (+0.77 kg CO2e/kg), and high process utility energy (+1.05 kg CO2e/kg). The natural origin HPC system offers a moderate mitigation pathway, benefiting from the inherent biogenic sequestration of cellulose (‐0.90 kg CO_2_e/kg), yet still results in a net positive footprint (+2.37 kg CO_2_e/kg) due to substantial raw material impacts (+1.76 kg CO2e/kg) and processing energy demands (+1.02 kg CO2e/kg).

**FIGURE 5:**
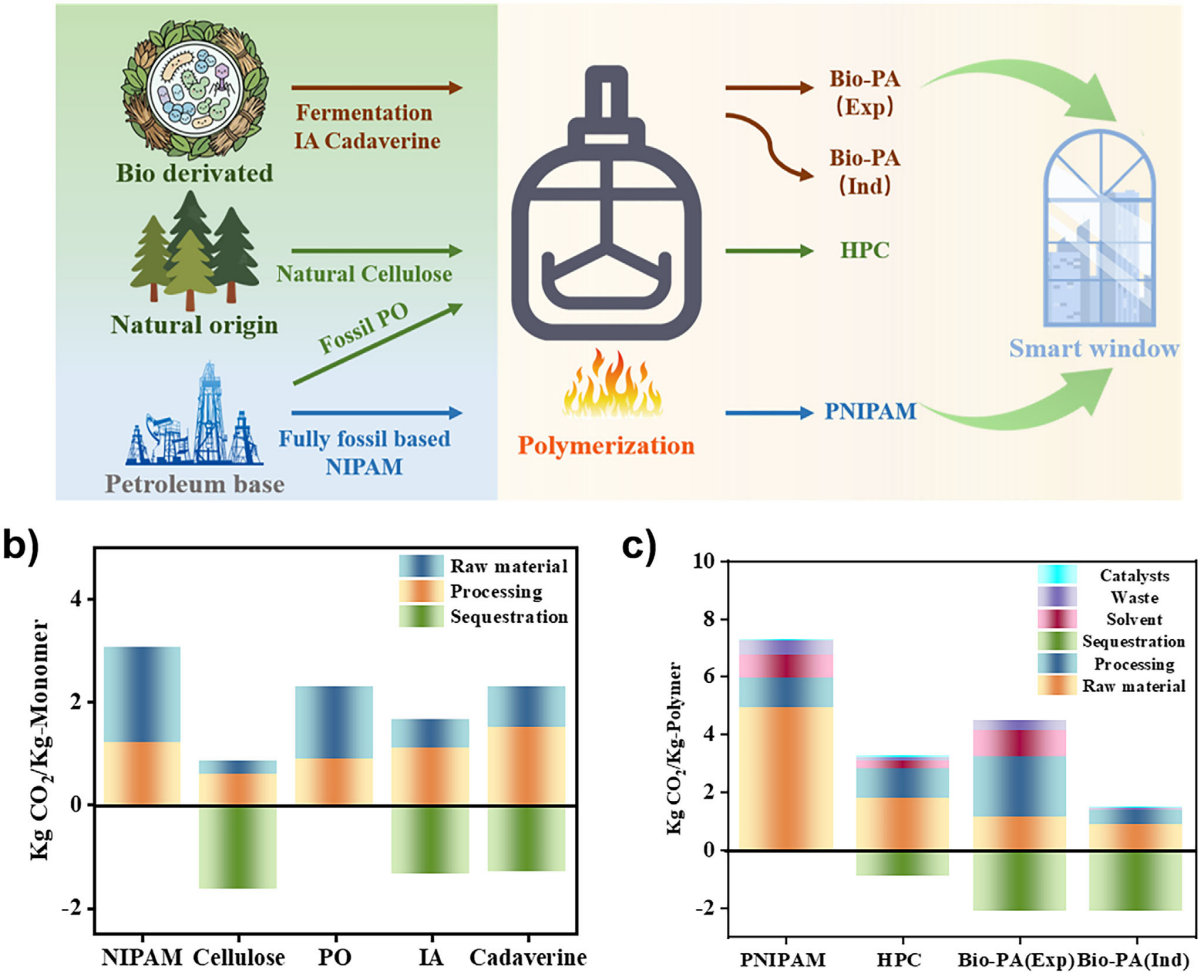
Life cycle carbon footprint of star‐branched polyamide versus conventional thermochromic polymers. (a) Schematic illustration of the polymerization process for different polymers from the monomer stage. (b) and (c) Life cycle greenhouse gas emissions of produce progress for the monomer and polymer.

In contrast, the Bio‐PA platform fundamentally breaks this linear reliance on fossil feedstocks by utilizing highly sequestering monomers like itaconic acid and cadaverine. Both of them achieve a low net carbon footprint (+0.30 and +1.01 kg CO_2_e/kg) because the massive biogenic carbon sequestration during biomass growth (−1.35 and −1.29 kg CO_2_e/kg) nearly offsets the emissions from its acquisition and processing. By integrating these bio‐derived monomers, the Bio‐PA system achieves an exceptional biogenic sequestration factor of −2.11 kg CO_2_e/kg. This demonstrates that shifting toward bio‐derived, star‐branched topologies can deliver climate‐positive material sourcing for thermochromic smart window applications, addressing the critical need for sustainable building materials. To evaluate the industrial and commercial viability of the Bio‐PA resin platform, we analyzed the scalability levers that reduce production emissions and energy costs from the bench to commercial scale. At the laboratory scale, the net carbon footprint of experimental Bio‐PA (Exp) remains relatively high (+2.36 kg CO_2_e/kg). This is primarily due to the energy‐intensive process utility stage (+2.07 kg CO_2_e/kg) and severe solvent makeup losses (+0.90 kg CO_2_e/kg), which reflects the inherent inefficiencies of small‐batch syntheses, unoptimized thermal management, and lack of solvent recovery typical in early‐stage material development. However, projecting this synthesis to an industrial scale reveals massive environmental and operational efficiency gains. Crucially, by transitioning to solvent‐free processing or implementing closed‐loop solvent recovery (reducing solvent losses to 0.00 kg CO_2_e/kg) and optimizing utility energy (+0.50 kg CO_2_e/kg). Because the gross emissions are entirely offset by the massive biogenic carbon sequestration (‐2.11 kg CO_2_e/kg), the net cradle‐to‐gate carbon footprint is driven down to an outstanding net‐negative value of −0.65 kg CO_2_e/kg. Achieving this absolute climate‐positive state demonstrates that shifting toward bio‐derived, star‐branched topologies can deliver transformative material sourcing for smart window applications. This net‐negative status can be further optimized through the progressive electrification of the biomass transportation network and the strategic switch from conventional fossil‐derived processing heat to sustainable, biomass‐derived thermal energy sources.

Beyond cradle‐to‐gate metrics, the holistic sustainability of these polymers depends heavily on their end‐of‐life (EoL) circularity (Figure S41). HPC, while possessing moderate biodegradability, is not amenable to conventional mechanical melting due to thermal degradation, meaning chemical recovery is economically non‐viable, and it is best managed via composting. PNIPAM faces severe recyclability constraints; it cannot be mechanically recycled in commercial post‐consumer sorting streams, and chemical depolymerization of its resilient carbon‐carbon backbone is highly energy‐intensive and cost‐prohibitive, leading to a disposal fate of incineration that releases fossil CO_2_ and NO_x_. Conversely, as a thermoplastic polyamide, Bio‐PA can be repeatedly re‐melted, extruded, and compounded, fitting seamlessly into existing mechanical nylon recycling streams (similar to PA56). Crucially, the amide linkages can undergo catalyzed acid/base hydrolysis or ammonolysis back into itaconic acid and cadaverine precursors at high selectivity, enabling true circular closed‐loop recycling. Furthermore, engineering polyamides used in durable goods retain biogenic carbon for long operational lifespans (10–20 years), maximizing their carbon storage impact.

To further evaluate commercial viability, we conducted a prospective Techno‐Economic Analysis (TEA) (Figure S41). Conventional PNIPAM is highly priced (>$80–$250/kg) due to the multi‐step synthetic complexity of the NIPAM monomer, restricting it to ultra‐high‐margin niches. Mature commodity‐specialty HPC costs approximately $8–$15/kg. While lab‐scale Bio‐PA synthesis is economically constrained by manual precipitation steps, at a commercial scale, utilizing bio‐itaconic acid (∼$1.50–$2.20/kg) and bio‐cadaverine (∼$2.50–$3.20/kg) brings polymer production costs to approximately $2.80–$4.00/kg. While this represents a 20–40% green premium over fossil‐based PA66 or PA6, it delivers cost parity with specialty engineering polyamides (e.g., PA11, PA12). Realizing these commercial projections requires overcoming specific scaling bottlenecks, specifically transitioning from solvent‐based precipitation to concentrated water‐based salts or reactive extrusion, which serves as the critical tipping point for achieving both economic viability and the projected net‐negative carbon footprint.

Critically, this analysis must account for the emerging global regulatory landscape regarding embodied carbon in building materials. Conventional PNIPAM‐based smart windows carry high embodied carbon penalties that will increasingly be subject to carbon compliance costs. HPC‐based smart windows exhibit a competitive carbon footprint, their further development is severely hindered by inherent structural limitations. The inability to precisely control their molecular weight and polymer architecture makes the fine‐tuning of their macroscopic thermochromic performance highly challenging. The scalable Bio‐PA platform virtually eliminates these environmental externalities. With a net‐negative carbon footprint (−0.65 kg CO_2_e/kg) alongside robust EoL circularity at industrial volumes, the Bio‐PA platform not only achieves superior structural and thermochromic performance but also translates directly into powerful economic incentives for the actual construction industry. Specifically, the drastic reduction in embodied carbon achieved by this material provides a clear commercial advantage which not only by reducing operating energy costs over the building's entire lifecycle, but also by generating premium carbon credits and maximizing certification value (Leadership in Energy and Environmental Design, Building Research Establishment Environmental Assessment Method, and Green Building Evaluation Labels) at the initial construction and material procurement stages. This provides a compelling market incentive for its long‐term industrial adoption in energy‐efficient architecture.

These results establish highly bio‐based 4W polyamides as a transformative platform for next‐generation ultrahigh concentration pseudosolid state smart windows, uniquely combining salt‐resistant stability, large‐temperature = range tunability, and dual‐mode electro‐thermal control, overcoming fundamental limitations of existing materials while enabling both passive and active building energy management.

## 3 Conclusion

Here, we report a novel design paradigm for stimuli‐responsive materials by precisely tailoring pyrrolidone‐containing star biopolyamides (2W–4W) with dynamic metal‐coordinating capabilities, enabling thermoresponsive phase separation within a pseudo‐solid matrix (water content is 50% or less). Based on a Nylon‐type skeleton synthesized from itaconic acid and aliphatic diamines, the backbone features repeating pyrrolidone and aliphatic units, where a cadaverine (C5) spacer optimizes the hydrophilic‐hydrophobic balance for thermoresponsiveness. Although increasing the molecular weight sharpens the coacervation, excessive molecular weights induce solution heterogeneity and restrict chain mobility, thus depressing the transition behavior. To overcome this trade‐off, we engineered a star‐shaped, highly branched architecture, where the four‐arm polyamides displayed distinct, high‐fidelity thermoresponsiveness. Crucially, *T*_c_ of the four‐arm polyamide was widely tunable (8–66°C) not only through structural metrics like molecular weight but also via the aqueous ionic composition. Notably, in contrast to traditional Na^+^‐driven salting‐out that drops the *T*_c_ to 8°C, Ca^2+^ incorporation triggered a dramatic salting‐in effect, significantly boosting both *T*_c_ and solubility. MD simulations and DFT calculations elucidated the underlying mechanism: the moderate coordination capacity of pyrrolidone dynamically traps Ca^2+^ ions without disrupting their hydration shells, effectively drawing water into the polymer network. Consequently, thermoresponsiveness was preserved even at an ultra‐high ionic strength of 5 mol/L. By rendering the light‐scattering coacervate droplets cationic, electrostatic repulsion stabilized the turbid state for over 8 h, while electrophoretic control over the spatial turbidity unlocked dual electro‐thermal responsiveness. This strategy successfully converted a polyamide insoluble in deionized water into a highly soluble, thermoresponsive pseudo‐solid system (50% water) upon Ca^2+^ addition. The fabricated smart window achieved exceptional macroscopic dispersion stability and superior cell‐temperature regulation over conventional thermoresponsive polymers. Overall, these ion‐coordinating star polyamides establish a paradigm‐shifting platform for next‐generation stimuli‐responsive soft systems, spanning implantable biosensors to battery‐driven smart windows.

## 4 Experimental Section

### 4.1 Materials

Itaconic acid was purchased from Macklin (> 99%). Cadaverine (98%) and Tris(2‐aminoethyl) amine (98%) were supplied by TCI. PAMAM dendrimer (0 generation) (97%), Hexafluoroisopropanol (HFIP) (99%), and Poly(N‐isopropylacrylamide) (PNIPAM) (*M*_w_∼100 000 g/mol) were ordered from Adamas. Ethanol, sodium hydroxide, and hydrochloric acid were supplied by Sinopharm Chemical Reagent; sodium chloride, calcium chloride, and Hydroxypropyl cellulose (HPC) (*M*_w_∼100 000 g/mol) were purchased from Aladdin.

### 4.2 Preparation of Salt Monomers

A solution of itaconic acid (1 mol) in EtOH (300 mL) was mixed with the solution of cadaverine (1 mol) in EtOH (200 mL) to get a 1:1 monomer mixture. The mixture was kept for 30 min at room temperature to form salts (94−97% yields). The salts were collected and dried at a certain temperature in a vacuum system. In ^1^H NMR spectra of salt monomer (Figure S1), characteristic signals (unit: ppm) were observed at H_d_ 1.36 (CH_2_CH_2_CH_2_ of cadaverine), H_e_ 1.61 (CH_2_CH_2_CH_2_CH_2_CH_2_ of cadaverine), H_f_ 2.95 (NH_2_CH_2_CH_2_CH_2_CH_2_CH_2_NH_2_ of cadaverine) and H_a_ 3.05 (CH_2_COOH of IA) together with 5.28 and H_b_ 5.30 (C = CH_2_ of IA) and H_c_ 5.75 (C = CH_2_ of IA). The integral value of the salt monomer also confirmed the 1:1 mixture ratio.

### 4.3 Preparation of Amino Acid Monomers and Oligomers

The dried salts were dissolved in 300 mL of H_2_O, and the monomer solution was added to the single‐neck round‐bottom flask. The monomer solution was heated at 110°C with the refluxing system for 24 h. Finally, the targeted components were collected after evaporation (95%–97% yields). In the ^1^H NMR spectrum of the amino acid monomer (Figure S2a), characteristic pyrrolidone signals (unit: ppm) were observed at H_a_ 3.45–3.62, H_b_ 3.12, H_c_ 2.50–2.63. The integral value of the amino acid monomer confirmed the structure of the amino acid monomer. The 215 m/z peak of the liquid chromatography–mass spectrometry (LC‐MS) spectrum also proved the preparation of the amino acid monomer (Figure S2b). Further, the amino acid monomers were heated for 2 h to prepare the amino acid oligomer at a certain temperature, as the onset temperature detected by thermogravimetry (TGA) and differential thermal analysis (DTA) (Figure S2c), accompanied by a weight loss corresponding to the weight percent of one water molecule compared with the salt monomer (two water molecules), which also confirmed that the amino acid monomer was synthesized successfully. For the oligomer, the typical amide peaks of ^1^H NMR and fourier transform infrared (FTIR) spectra revealed the complete synthesis of the oligomer (Figure S3).

### 4.4 Synthesis of Star‐Polyamides

The amino acid oligomer from the salt monomer and individual branch center were mixed as the feed ratio and agitated in the presence of a protective atmosphere of Ar, to be polymerized in bulk at 180°C, where the reaction temperature was set to the same as the onset weight loss temperature of the TGA curve of amino acid monomers. For instance, the amino acid monomer with tris(2‐aminoethyl) amine was put in a sealed tube equipped with a mechanical stirrer bar and heated to around 180°C under slow agitation and Ar flowing for 4−8 h to control molecular weight. The final polymers (yields 88−92% for 2 W, 3 and 4 W) were washed with ethanol 3 times to remove the remaining monomers and then characterized by ^1^H NMR and ^13^C NMR, FTIR, and gel permeation chromatography (GPC).

## Author Contributions

**Jie Liu**: conceptualization, writing – original draft, methodology, data curation, validation, visualization, formal analysis, investigation. **Xiaotian Zhu**: data curation. **Jinjian Hou**: writing – review and editing, software. **Jinze Du**: software, writing – review and editing, funding acquisition. **Mohammad Asif Ali**: conceptualization, writing – review and editing, methodology. **Maiko K. Okajima**: conceptualization, writing – review and editing. **Tatsuo Kaneko**: conceptualization, writing – review and editing, supervision, funding acquisition, project administration, software, resources.

## Funding

The National Natural Science Foundation of China (NSFC) research fund for International Senior Scientists (RFIS‐III) (Grant number W2531033), the Fundamental Research Funds for the Central Universities (JUSRP202416002), and Ningbo Natural Science Foundation (Grant number 2023J393).

## Conflicts of Interest

The authors declare no conflicts of interest.

## Supporting information




**Supporting File 1**: advs78227‐sup‐0001‐SuppMat.docx.

**Supporting File 2**: advs78227‐sup‐0002‐VideoS1.mp4.

**Supporting File 3**: advs78227‐sup‐0003‐VideoS2.mp4.

**Supporting File 4**: advs78227‐sup‐0004‐VideoS3.mp4.

**Supporting File 5**: advs78227‐sup‐0005‐VideoS4.mp4.

**Supporting File 6**: advs78227‐sup‐0006‐VideoS5.mp4.

**Supporting File 7**: advs78227‐sup‐0007‐VideoS6.mp4.

## Data Availability

The data that support the findings of this study are available from the corresponding author upon reasonable request.
