# Supplementary material for: Ionically Counter‐Programmable Phase‐Separation of Superstable Star‐Biopolyamide Coacervates for Electrothermally‐Responsive Pseudosolid Smart Windows
Source: Adv Sci (Weinh). 2026 Oct 11:e78227. Online ahead of print. doi: 10.1002/advs.78227 (PMC13655256; doi:10.1002/advs.78227)
Supplement: Supplementary file 1 [file ADVS-9999-e78227-s007.docx]

*Supporting Information*

**Ionically Counter-programmable Phase-separation of Superstable Star-biopolyamide Coacervates for Electrothermally-Responsive Pseudosolid Smart Windows**

Jie Liu^a^, Xiaotian Zhu^a^, Jinjian Hou^b^, Jinze Du^c,d^, Mohammad Asif Ali^a,^*, Maiko K. Okajima^a,^*, and Tatsuo Kaneko^a,^*

^a^The Key Laboratory of Synthetic and Biological Colloids, Ministry of Education, School of Chemical and Material Engineering, Jiangnan University, Wuxi 214122, China.

^b^ School of Chemistry and Chemical Engineering, Hainan University, Haikou 570228, China

^c^School of Chemical Engineering, Tianjin University, Tianjin 300072, China

^d^Zhejiang Institute of Tianjin University, Ningbo 315000, China

**Table of Contents**

**1 Instruments and measurements**

**1.1 Fourier transform infrared (FTIR) spectroscopy**

**1.2 Nuclear magnetic resonance (NMR)**

**1.3 Thermal Analysis System TGA/DTA (TGA/DSC 3+)**

**1.4 Liquid Chromatography-Mass Spectrometry (LC-MS)**

**1.5 Gel permeation chromatography (GPC)**

**1.6 Size Exclusion Chromatography-Multi-Angle Light Scattering (SEC-MALS)**

**1.7 Optical microscope**

**1.8 Ultraviolet-visible spectrum (UV-Vis)**

**1.9 Dynamic light scattering (DLS) and Zeta-potential**

**1.10 Low-Field Nuclear Magnetic Resonance (LF-NMR)**

**1.11 X-ray photoelectron spectroscopy (XPS)**

**1.12 Thermo-responsive behavior stability**

**1.13 Smart window outdoor simulation**

**1.14 Density functional theory (DFT)**

**1.15 Molecular dynamics simulation (MD)**

**1.16 Life Cycle Assessment (LCA)**

**2. Supplementary figures and tables**

**1 Instruments and measurements**

**1.1** **Fourier transform infrared (FTIR) spectroscopy**

The test was measured by using Thermo Fisher Nicolet iS50. All samples' data were determined using the ATR attachment of the instrument. The FTIR data is corrected and processed by the software OMNIC, and the Origin is used for drawing.

Temperature-dependent FTIR were measured from 25 to 60°C

**1.2 Nuclear magnetic resonance (NMR)**

The samples were dissolved in deuterated water and loaded into an NMR tube. NMR measurements and temperature-dependent NMR (25-60°C) were conducted on an AVANCE NEO 600MHz, Bruker, and the resulting ^1^HNMR spectra were analyzed using MestReNova software. Data processing and plotting were performed with Origin software.

**1.3** **Thermal Analysis System TGA/DTA (TGA/DSC 3+)**

The test was carried out using a METTLER TOLED TGA/DSC 3+. Samples were first heated to dry at 120 ℃ and held at this temperature for 30 min under a nitrogen atmosphere to remove most of the absorbed water. The samples were then cooled to 30 ℃, followed by heating the samples from 30 to 800 ℃ at a scanning rate of 10 k/min under nitrogen.

**1.4 Liquid Chromatography-Mass Spectrometry (LC-MS)**

The sample was dissolved in ultrapure water. LC-MS measurements were conducted on a MALDI SYNAPT MS, Waters, and the resulting LC-MS spectra were analyzed by using MassLynx software.

**1.5 Gel permeation chromatography (GPC)**

The samples were dissolved in HFIP + 0.02 M NaTFAc. GPC measurements were conducted on an PL-GPC50 Agilent with the two columns are PL HFIPgel, 7.5 × 300 mm, 9 μm. The flowing speed was 1.0 mL/ min. The measurements were obtained 100 μL sample solutions at 40 °C. The resulting spectra were analyzed with standard sample PMMA by using Lab solution software, and the Origin is used for drawing.

**1.6 Size Exclusion Chromatography-Multi-Angle Light Scattering (SEC-MALS)**

The samples were weighed and dissolved in DMF, followed by filtration through a 0.22 μm PTFE filter. SEC-MALS measurements were conducted on an Agilent PL-GPC 50 instrument equipped with two PLgel Mixed-B columns (8 μm). DMF containing 0.05 M LiBr was used as the mobile phase at a flow rate of 1.0 mL/min. A volume of 100 μL of the sample solution was injected, and the measurements were performed at a column temperature of 45 °C with an analysis time of 30 min. The system was coupled with a refractive index (RI) detector and a laser light scattering detector. The absolute molecular weights were subsequently calculated based on the sample concentrations.

**1.7 Optical microscope**

Polymer aqueous solutions (2.0 wt%) were dispersed on the glass slide, and the electro responsive progress was observed under the voltage provided by DC power supply SSG-305DS, A-BF with two platinum electrodes (Φ 0.5 * 37 mm). Microscope images of the samples were obtained using MP41 (Mshot) with eye lens (WF10X/22) and objective lens (PL 60/0.70).

**1.8** **Ultraviolet-visible spectrum (UV-Vis)**

The temperature-dependent UV-vis spectra of all the samples were measured using a UV-2700 (Shimadzu), and the sample chamber was covering with temperature-controlled holder. The temperature-controlled support is connected to a temperature-controlled Peltier (S-1700, Shimadzu), with temperature range of 3-80 °C.

The electro responsive UV-vis spectra were also measured by same UV-2700, in the meanwhile the two platinum electrodes were putting into the sample cells connected with DC supply as mentioned above.

**1.9 Dynamic light scattering (DLS) and Zeta-potential**

90Plus PALS (Bruker) was used to perform Zeta-potential of sample solution and particle size analysis of different temperatures, with a temperature range of 3-75 °C. Three tests were performed, and the average value was taken.

**1.10 Low-Field Nuclear Magnetic Resonance (LF-NMR)**

The transverse relaxation time (*T*_2_) of the polyamide liquid samples was determined using a MicroMR12-025V low-field NMR analyzer (Niumag Analytical Instrument Co., Suzhou, China). The instrument operated at a proton resonance frequency of 12 MHz. The relaxation data were acquired utilizing a Carr-Purcell-Meiboom-Gill (CPMG) pulse sequence. The specific acquisition parameters for the measurements were configured as follows: the 90° pulse width (P1) and 180° pulse width (P2) were set to 6.8 μs and 10.8 μs, respectively; the spectral width (SW) was 125 kHz; the repetition waiting time (TW) was 6500 ms; the number of scans (NS) was 8; the echo time (TE) was 1.2 ms; and the number of echoes (NECH) was set to 18,000. All measurements were conducted under stable room temperature conditions.

**1.11 X-ray photoelectron spectroscopy (XPS)**

ESCALAB Qxi (Thermo Fisher) was used for element analysis of samples, the sample solution after freeze drying then performed the measurement. The x-ray source type was monochromated Al Kα radiation,and the full survey was conducted with the range of -100-1350 eV, pass energy (100 eV) and energy step size (1 eV). The accurate element scans were shown below: N 1s 392-410 eV, O 1s 525-545 eV, pass energy (20 eV) and energy step size (0.1 eV).

**1.12 Thermo-responsive behavior stability**

Two types of ion solution (NaCl and CaCl_2_) (1.0 M) and aqueous solution of three types LCST polymers (4W, PNIPAM, HPC) (1.0 wt%) were placed in the dry oven at a certain temperature (> T_c_). Then the stability of each condition were defined by the settlement time from turbid sate to transparent state.

**1.13 Smart window outdoor simulation**

Outdoor simulated tests were performed by using boxes made of polymethyl methacrylate (PMMA) board (14.5 × 9.5 × 12 cm, board thickness 0.8 cm) and three test compartments (16 × 11 × 0.5 cm, board thickness 0.5 cm), a smart window was fabricated by sandwiching the 4W solution between two pieces of glasses. Meanwhile, two control compartments were equipped with the same bilayer glass with air and H_2_O in between. Temperature detector JAALEE (China) was applied to detect the indoor temperatures in each box. The tests were conducted in Wuxi to evaluate the temperature regulation ability of 4W smart window. Simulation test setup was settled outdoor without any shelter and subjected to direct sunlight. Temperature monitoring for

continuous 10 h (from 8:00 a.m. to 18:00 p.m.) was recorded and the sampling interval was 1 min.

Outdoor simulation measurements were standardized to an average irradiance of 739 W/m^2^ from database NASA POWER Data Access Viewer (https://power.larc.nasa.gov/data-access-viewer). Humidity: The relative humidity during testing was tightly monitored and reported at a standardized 53.1±5.2%. Device Dimensions: The tested devices were standardized to an active area of 10×15 cm^2^.

**1.14 Density functional theory (DFT)**

The coordination molecular models of 4W, H_2_O and metal ions were constructed using Materials Studio, with atoms represented as follows: H (white), N (blue), C (gray), O (red), Ca (green), and Na (purple). Atomic charges were assigned accordingly. Energy minimization was performed using the Forcite module with the COMPASS forcefield, under a system temperature of 298 K and 500 iteration steps. Based on the optimized molecular model, energy calculations were carried out via the Forcite module, again using the COMPASS forcefield.[1]

**1.15 Molecular dynamics simulation (MD)**

Quantum chemistry calculations were first performed to optimize molecular geometries of molecules (MOL) were used the Gaussian 16 package [2] at B3LYP/6-311+G(d) level of theory. The atomistic force field parameters for all are described by the OPLS-AA [3].

In system 1, 200 CaCl2, 1000 MOL, 5556 H2O were randomly inserted into a simulation box which is x = 7.73 nm, y = 7.73 nm, and z =7.73 nm, finally.

Atomistic simulations were performed using GROMACS package [4] with cubic periodic boundary conditions. The equations for the motion of all atoms were integrated using a classic Verlet leapfrog integration algorithm with a time step of 1.0 fs. A cutoff radius of 1.4 nm was set for short-range van der Waals interactions and real-space electrostatic interactions. The particle-mesh Ewald (PME) [5] summation method with an interpolation order of 4 and a Fourier grid spacing of 0.12 nm was employed to handle long range electrostatic interactions in reciprocal space. In all the three directions, periodic boundary conditions were imposed. Leapfrog algorithm was used to integrate the Newtonian equation of motion. The MD simulation was processed in an NPT ensemble and the simulation time all is 40 ns. Then the temperature was maintained by the V-rescale thermostat at 298.15 K. the pressure was maintained at 1 bar by the Berendsen barostat in an isotropic manner.

**1.16 Life Cycle Assessment (LCA)**

A cradle-to-grave life cycle assessment (LCA) was performed using Carbon AI (carbonai.carbonstop.com; Carbonstop Co., Ltd.) in compliance with ISO 14067:2018, the GHG Protocol, and the ISO 14064 series of standards. The assessment compared the global warming potential (GWP) of two conventional thermochromic polymer and experimental system and industrial system of Bio-PA including raw material, processing and sequestration. The compiled datasets were analyzed to establish the net carbon emission profiles for each scenario.

[1] Li, J., Liang, J., Zhao, C., Feng, N., Guo, G., Zhao, X., Zhou, Z.,Sun, H. *Int. J. Hydrogen Energy* 2025, *149*, 149999.

[2] M. J. Frisch, G.W. Trucks, H.B. Schlegel, G.E. Scuseria, M.A. Robb, J.R. Cheeseman, G. Scalmani, V. Barone, G.A. Petersson, H. Nakatsuji, X. Li, M. Caricato, A.V. Marenich, J. Bloino, B.G. Janesko, R. Gomperts, B. Mennucci, D.J. Hratch. Gaussian 16, Revision C.01 Gaussian, Inc., Wallingford CT (2016).

[3] W.L. Jorgensen, D.S. Maxwell, J. Tirado-Rives, Development and Testing of the OPLS All-Atom Force Field on Conformational Energetics and Properties of Organic Liquids, J. Am. Chem. Soc. 118 (45) (1996) 11225–11236.

[4] Páll Szilárd, Mark James Abraham, Carsten Kutzner, Berk Hess, Erik Lindahl. Tackling Exascale Software Challenges in Molecular Dynamics Simulations with GROMACS. 2015, arXiv:1506.00716.

[5] Darden T., York D., Pedersen L. Particle mesh Ewald: An N⋅log(N) method for Ewald sums in large systems. The Journal of Chemical Physics 98, 10089-10092 (1993).

**2. Supplementary figures and tables**

**Scheme S1.** Syntheses of star-polyamide with pyrrolidone-containing arms. a) Syntheses routine of pyrrolidone oligomers from amino acid monomer by salt monomer method; b) Architectural control method of diamino (2W), triamino (3W), and tetraamino (4W) star-polyamides from amino acid oligomer and various branch center.

**Figure S1.** a) ^1^H-NMR spectrum of salt monomer from itaconic acid and cadaverine;

b) FTIR spectra of salt monomer, itaconic acid and cadaverine

**Figure S2.** a) ^1^H-NMR spectrum of amino acid monomer from salt monomer; b) LC-MS spectrum of amino acid monomer; c) Thermogravimetric analysis (TGA) and differential thermal analysis (DTA) curves of the salt monomer and amino acid monomer

**Figure S3.** a) ^1^H-NMR spectrum of oligomer from amino acid monomer;

b) FT-IR spectra of amino acid monomer and oligomer

**Figure S4.** FTIR spectra of 2W, 3W and 4W star-polyamides

**Figure S5.** GPC curves of 2W, 3W and 4W star-polyamides

**Figure S6.** NMR spectra of star-polyamides, 2W, 3W and 4W, a) ^1^H-NMR, b) ^13^C-NMR

**Figure S7.** Integral spectra of ^1^H and ^13^C NMR for 2W

**Figure S8.** Integral spectra of ^1^H and ^13^C NMR for 3W

**Figure S9.** Integral spectra of ^1^H and ^13^C NMR for 4W

**Figure S10.** SEC-MALS curves of 2W, 3W and 4W star-polyamides

**Figure S11.** a) Apparent viscosity as a function of shear rate for the 4W sample at various molar concentrations (ranging from 0.75 M to 2.00 M) compared with a pure water (H_2_O) control. b) Plot of reduced viscosity (orange) and inherent viscosity (green) versus polymer concentration. The intrinsic viscosity [η] is determined to be 1.83 ± 0.14 mL/g by extrapolating the linear fits to zero concentration.

**Figure S12.** a) Thermogravimetric analysis (TGA) curves of the 2W, 3W, and 4W samples from 30 to 800 °C. b) Differential scanning calorimetry (DSC) thermograms of the corresponding samples, showing their thermal transition behaviors and endothermic peaks over a temperature range of 40 °C to 200 °C.

**Figure S13.** A 3D plot illustrating the particle size distribution by volume percentage across a temperature range from 25 °C to 45 °C. The graph demonstrates a clear temperature-responsive behavior, where the peak of the size distribution shifts significantly toward larger hydrodynamic diameters (Size in μm) as the temperature increases from 25 °C (red curve) to 45 °C (yellow curve).

**Table S1.** Molecular structure of the different molecular weight 4W polymers

|  | *M*_n_(g/mol) | *M*_w_(g/mol) | PDI | *T*_c_ (2%) |
| --- | --- | --- | --- | --- |
| Oligomer | 4800 | 6750 | 1.41 | N/A |
| 4W -1 | 4980 | 11400 | 2.30 | 51 |
| 4W -2 | 6760 | 20500 | 3.00 | 43 |
| 4W -3 | 6840 | 24700 | 3.60 | 38 |
| 4W -4 | 8250 | 25300 | 3.06 | 33 |
| 4W -5 | 9200 | 31100 | 3.38 | 25 |

Number-average molar mass (*M*_n_ GPC), weight-average molar mass (*M*_w_ GPC), and polymer dispersity index (Đ) estimated by GPC using HFIP as the eluent; Cloud temperature of polymer determined by UV–vis spectroscopy

**Figure S14.** GPC curves for 4W-1 to 4W-5 star-polyamides

**Figure S15.** a) Photographs of 4W-1 to 4W-5 branched polyamide aqueous solution below and above the cloud temperature; b) Polymer concentration-dependent cloud temperature curves of different branched polymer aqueous solutions formed from 4W-1 to 4W-5; c) Temperature-dependent transmittances of 4W-1 to 4W-5 polymer aqueous solutions; d) Diameters of 4W-1 to 4W-5 polymer aqueous solutions at different temperatures.

**Figure S16.** Diameter distributions of 4W-1 to 4W-5 polymer aqueous solutions at different temperatures

**Figure S17.** Temperature-depend Integral^1^H-NMR spectra from 25 to 30 °C

**Figure S18.** Temperature-depend Integral^1^H-NMR spectra from 35 to 45 °C

**Figure S19.** Temperature-depend Integral^1^H-NMR spectra from 50 to 60 °C

**Figure S20.** Time-domain signal amplitude distribution profile for the 4W sample from 0.01 ms to 10000 ms.

**Figure S21.** Temperature-dependent FT-IR spectra of the 4W solution upon heating from 25 to 45 °C

**Figure S22.** 2Dcos synchronous and asynchronous spectra.

**Figure S23.** Salt addition effects on clouding behavior of aqueous solutions for star polyamides 2W, 3W, and 4W.

a, b) Temperature-dependence of transmittances under different concentrations of NaCl and CaCl_2_; c) Clouding temperature, Tc, in various conditions of salt addition. c, d) Coacervate sizes at different temperatures with different concentrations of NaCl and CaCl_2_, respectively. e) Tc change by CaCl2 addition with different ionic strengths; f) Competitive ion experiments of 4W aqueous solution with different ion conditions.

**Figure S24.** Microscopic images of 4W in CaCl_2_ above *T*_c_

Table S2. The *ζ* -potential of 2W, 3W and 4W with different condition

| *ζ* (mV) | Pure  solution | NaCl  (0.5M) | CaCl_2_  (0.5M) |
| --- | --- | --- | --- |
| 2W | 4.76±0.24 | 1.95±0.34 | 6.93±0.15 |
| 3W | 6.34±0.25 | 3.48±0.16 | 9.49±0.35 |
| 4W | 9.45±0.14 | 6.26±0.21 | 18.39±0.36 |

*ζ−*potential of polymer aqueous solution with a concentration of 2 wt% at 50 °C was determined by DLS analysis

Table S3. The *ζ* -potential of 2W, 3W and 4W with different pH

| *ζ* (mV) | pH=3 | pH=5 | pH=7 | pH=9 | pH=11 |
| --- | --- | --- | --- | --- | --- |
| 2W | 15.39±0.35 | 9.85±0.34 | 4.85±0.37 | -1.29±0.44 | -9.32±0.48 |
| 3W | 16.03±0.36 | 10.38±0.16 | 6.44±0.22 | -1.55±0.21 | -9.87±0.33 |
| 4W | 16.48±0.24 | 11.04±0.21 | 9.39±0.27 | -1.41±0.47 | -9.93±0.36 |

**Figure S25.** Molecular simulation of Na with pyrrolidone polymer and H_2_O.

**Figure S26.** Molecular simulation of Ca with pyrrolidone polymer and H_2_O.

Table S4. RDF result of Ca with H_2_O and pyrrolidone molecule

|  | First peak distance(Å) | Peak height | CN (3.0Å) |
| --- | --- | --- | --- |
| RDF-H_2_O-O-Ca | 2.34 | 23.53 | 3.668 |
| RDF-MOL-N-Ca | 2.42 | 2.88 | 0.175 |
| RDF-MOL-O-Ca | 2.26 | 22.38 | 1.438 |

H_2_O-O-Ca represents the RDF of Ca with O in the H_2_O

MOL-O-Ca represents the RDF of Ca with O in the pyrrolidone from molecule

**Figure S27.** Molecular dynamics of different time at a) 0 ns, b) 10 ns, c) 30 and d) 40 ns.

**Figure S28.** Molecular dynamics of all the system at a) beginning and b) ending.

**Figure S29.** Time-domain signal amplitude distribution profile for the 4W-Ca samples from 0.01 ms to 10000 ms.

**Figure S30.** Polymer settlement time of different conditions with PNIPAM, HPC, and 4W polymer.

**Figure S31.** Stability test of 4W, PNIPAM and HPC with CaCl_2_ before and after 8h.

**Figure S32.** Optical picture of high concentration solution at 35℃ on the different time

**Figure S33.** Optical picture of high concentration solution with different angles upon *T*_c_

**Figure S34.** a) Schematic illustration detailing the temperature-responsive phase transition of a branched polyamide in an aqueous solution, mapping the dynamic balance of polymer-water hydrogen bonding, intermolecular hydrogen bonding, and ion coordination as the system moves above and below its cloud point temperature. b) Rheological properties of the pseudosolid smart window, illustrating the evolution of the storage modulus (G') and loss modulus (G'') as a function of temperature from 35 °C to 50 °C. c) Curve of shear stress against shear strain for the pseudosolid smart window with the solid-like material's yield point. d) Graph displaying shear strain versus time for the pseudosolid smart window, demonstrating its time-dependent deformation behavior.

**Figure S35.** Cyclic stability and optical reversibility of the 4W for 30 cycles.

**Figure S36.** a) Integral spectra of ^1^H NMR for 4W after 100 cycles

b) Time-dependent transmittances of 4W aqueous solution under the 10V.

**Figure S37.** Photos of the outdoor simulation test setup with a test compartment, a 4W smart window, an H_2_O window, and control with a normal window at 18:00 after rain.

**Life Cycle Assessment**

Carbon AI (carbonai.carbonstop.com, Carbonstop Co., Ltd., China) was employed to assist in data

collection for conducting a life cycle assessment (LCA) of HPC, PNIPAM, and Bio-PA. By issuing instructions to Carbon AI, the amounts of carbon dioxide (CO₂) emitted or saved during each process stage of preparation of the polymers were obtained. Subsequently, the amounts of CO₂ emitted during the polymerization were quantified.

The following are the instructions issued to the AI and the corresponding responses.

**Figure S38.** Instruction 1 and the corresponding responses

**Figure S39.** Instruction 1 and the corresponding responses

**Question1:** In the current preparation process of NIPAM (monomer of PNIPAM)，Cellulose and Propylene Oxide (monomer of HPC) and IA and Cadaverine, what is the average volume of carbon emissions or carbon abatement in each stage for these five monomer? It is required to provide specific numerical values for each individual step (e.g., raw material acquisition, production or processing, carbon sequestration during monomer preparation, etc.) and clearly indicate the data sources for each value. Please answer in English.

**Answer1:** Hello, I am Xiao Tan, an AI application developed by Carbonstop, specializing in carbon accounting and consultancy.

Regarding your inquiry about the specific carbon emission or abatement values for NIPAM, Cellulose, Propylene Oxide, Itaconic Acid (IA), and Cadaverine across their preparation stages, here is the detailed analysis based on available industry data and carbon accounting principles.

1. NIPAM (N-Isopropylacrylamide)

NIPAM is typically synthesized via the reaction of acrylonitrile with isopropyl alcohol or through the reaction of acryloyl chloride with isopropylamine.

Raw Material Acquisition: ~1.85 kg CO2e/kg. This involves the energy-intensive production of acrylonitrile and isopropylamine.

Production/Processing: ~1.20 kg CO2e/kg. This includes the chemical synthesis process, cooling, and purification (distillation).

Carbon Sequestration: 0 kg CO2e/kg. As a fossil-based monomer, it does not sequester biogenic carbon.

Data Source: Ecoinvent Database / Industry LCA reports for specialty monomers.

2. Cellulose (Natural Polymer/Monomer Source)

Cellulose is a biogenic material, usually derived from wood pulp or cotton.

Raw Material Acquisition (Forestry/Harvesting): ~0.25 kg CO2e/kg. Includes machinery fuel and transport.

Production/Processing (Pulping): ~0.60 kg CO2e/kg. Chemical pulping (Kraft process) requires significant energy, though often offset by black liquor recovery.

Carbon Sequestration: -1.63 kg CO2e/kg. Calculated based on the carbon content of cellulose ($C_6H_{10}O_5$).

Calculation: Carbon content $\approx 44.4\%$. $0.444 \times (44/12) \approx 1.63$.

Data Source: IPCC Guidelines for National GHG Inventories / FEFCO.

3. Propylene Oxide (PO)

Commonly produced via the HPPO process (Hydrogen Peroxide to Propylene Oxide) or the POSM process.

Raw Material Acquisition: ~1.40 kg CO2e/kg. Primarily from propylene and hydrogen peroxide production.

Production/Processing: ~0.90 kg CO2e/kg. Energy used in the oxidation reaction and separation.

Carbon Sequestration: 0 kg CO2e/kg.

Data Source: PlasticsEurope Eco-profiles.

4. Itaconic Acid (IA)

IA is a bio-based monomer produced via the fermentation of carbohydrates (like glucose) by Aspergillus terreus.

Raw Material Acquisition (Glucose/Corn Starch): ~0.55 kg CO2e/kg. Includes agricultural emissions (fertilizers, land use).

Production/Processing (Fermentation & Purification): ~1.10 kg CO2e/kg. High energy demand for aeration in fermenters and downstream crystallization.

Carbon Sequestration: -1.35 kg CO2e/kg. Based on the biogenic carbon fixed in the IA molecule ($C_5H_6O_4$).

Calculation: Carbon content $\approx 46.1\%$. $0.461 \times (44/12) \approx 1.69$. However, accounting for losses in the fermentation cycle, the net sequestration in the final monomer is typically cited around -1.35 to -1.50.

Data Source: Journal of Cleaner Production / Bio-based industry LCA studies.

5. Cadaverine (1,5-Diaminopentane)

Bio-based cadaverine is produced via the decarboxylation of lysine (derived from sugar fermentation).

Raw Material Acquisition (Lysine/Sugar): ~0.80 kg CO2e/kg.

Production/Processing: ~1.50 kg CO2e/kg. The enzymatic or microbial conversion and subsequent distillation are energy-intensive.

Carbon Sequestration: -1.29 kg CO2e/kg. Derived from the biogenic origin of the carbon skeleton ($C_5H_{14}N_2$).

Calculation: Carbon content $\approx 58.8\% \times (44/12)$ relative to the glucose input.

Data Source: Corporate Sustainability Reports (e.g., Cathay Biotech) / Bio-chemical engineering LCAs.

Summary Table (kg CO2e per kg of monomer)

Note: Negative values indicate carbon removal/abatement from the atmosphere.

Calculation Logic & Methodology:

1. Biogenic Carbon: For IA and Cadaverine, we calculate the carbon content of the molecule. For example, if a molecule is 40% carbon by mass, the sequestration is calculated as:

$1 \text{ kg Carbon} \times (44/12) = 3.67 \text{ kg } CO_2$. Adjusting for yield and process losses, the net sequestration at the raw material stage is roughly -1.5 to -1.8 kg $CO_2e$.

2. Energy Allocation: For production stages, we apply regional electricity grid factors (e.g., China's average grid factor or specific regional mixes) to the kilowatt-hours consumed during fermentation or synthesis.

3. Secondary Data: Where primary site data is unavailable, we utilize secondary data from commercial databases (like Ecoinvent or GaBi) to ensure the granularity of the chemical precursors is captured.

If you require a more specific calculation based on your actual utility bills or specific supplier data, I can provide a customized carbon accounting template for your facility.

**Figure S40.** Instruction 2 and the corresponding responses

**Figure S41.** Instruction 2 and the corresponding responses

**Figure S42.** Instruction 2 and the corresponding responses

**Question2:** Now according to these monomer carbon footprint value, then for HPC and PNIPAM according to their polymerization condition. And for IA and Cadaverine based polyamide our experimental system is first in ethanol for salt preparation second is 110 ℃ for 24 h amino acid monomer finally polymerization is 180 ℃ for 2h, another condition is to refer the industrial system. Please calculate the average carbon emissions for obtaining different polymers (HPC, PNIPAM, Bio-PA(Exp) and Bio-PA(Ind)) in different system providing specific numerical values for each step (e.g., raw material acquisition, production or processing , carbon sequestration during monomer preparation, solvent, catalyst, waste streams, etc.) and clearly indicating the data sources for each value End‑of‑life recyclability and techno‑economic analysis should be discussed to strengthen sustainability arguments. Please answer in English.

**Answer2:** Hello! I am Xiao Tan, an AI carbon consultant developed by Carbonstop. To provide a rigorous, comprehensive cradle-to-gate Life Cycle Assessment (LCA) for the four polymer pathways—H Poly(N-isopropylacrylamide) (PNIPAM), Bio-PA (Experimental), and Bio-PA (Industrial)—we must expand the system materials and utility heat. This expanded evaluation incorporates:

1.Upstream Monomers & Functional Reagents

2.Catalysts & Initiators

3.Solvent Makeup & Losses (accounting for recycling efficiency and emission factors of makeup solvents)

4.Process Utilities (electricity, direct heating, vacuum systems)

5.Waste Treatment & Effluent Management (hazardous waste incineration, wastewater treatment)

6.Biogenic Carbon Uptake/Sequestration Credits (for bio-based carbon permanently incorporated into the polymer backbone)

Below is the step-by-step calculation, followed by an end-of-life (EoL) recyclability review and a techno-economic analysis (TEA).

1. **Step-by-Step LCA Calculations**

Note: The emission factor used for general grid electricity is assumed at a standard baseline of 0.45 kg $\text{CO}_2\text{e/kWh}$, and process steam/thermal energy from natural gas at 0.06 kg $\text{CO}_2\text{e/MJ}$ (approx. 0.22 kg $\text{CO}2\text{e/kWh}{\text{th}}$).

1. HPC (Hydroxypropyl Cellulose)

HPC is synthesized via the etherification of alkali cellulose using propylene oxide in an organic solvent/aqueous suspension.

Step 1: Raw Material Acquisition (Backbone & Etherification Agent)

Dissolving wood pulp (Cellulose content ~0.55 kg/kg HPC): $0.55 \times 0.65\text{ kg CO}_2\text{e/kg} = 0.36\text{ kg CO}_2\text{e}$. (Data source: Ecoinvent v3.8 - Chemical pulp)

Propylene Oxide (PO, ~0.50 kg/kg HPC): $0.50 \times 2.80\text{ kg CO}_2\text{e/kg} = 1.40\text{ kg CO}_2\text{e}$. (Data source: PlasticsEurope eco-profile for propylene oxide)

Subtotal Feedstocks: $1.76\text{ kg CO}_2\text{e/kg}$

Step 2: Biogenic Carbon Sequestration

Incorporates biogenic cellulose backbone (~0.55 kg cellulose equivalent): $-0.55 \times 1.63\text{ kg CO}_2/\text{kg} = -0.90\text{ kg CO}_2\text{e/kg}$. (Data source: IPCC Guidelines for National GHG Inventories)

Step 3: Catalyst & Auxiliary Chemicals

Sodium hydroxide ($\text{NaOH}$, 50% solution, ~0.08 kg/kg HPC): $0.08 \times 1.10\text{ kg CO}_2\text{e/kg} = 0.09\text{ kg CO}_2\text{e/kg}$. (Data source: World Chlorine Council / Euro Chlor)

Step 4: Solvents (Isopropanol/tert-Butanol + Water)

Suspension medium: Isopropanol (IPA). Assuming 95% closed-loop recovery with a 5% makeup requirement ($0.15\text{ kg}$ makeup IPA/kg HPC): $0.15 \times 1.85\text{ kg CO}_2\text{e/kg} = 0.28\text{ kg CO}_2\text{e/kg}$. (Data source: Ecoinvent - Isopropanol production)

Step 5: Process Energy (Alkalization, Etherification, Solvent Distillation, Drying)

Electricity (Stirring, drying, centrifugation): $1.2\text{ kWh/kg} \times 0.45\text{ kg CO}_2\text{e/kWh} = 0.54\text{ kg CO}_2\text{e/kg}$.

Thermal energy (Solvent recovery distillation & reactor jacket heating): $8\text{ MJ/kg} \times 0.06\text{ kg CO}_2\text{e/MJ} = 0.48\text{ kg CO}_2\text{e/kg}$. (Data source: Chemical Engineering Plant Cost & Energy Benchmarks)

Step 6: Waste Stream Treatment

Neutralization salt effluent & organic COD load in wastewater treatment plant (WWTP): $0.12\text{ kg CO}_2\text{e/kg}$. (Data source: Ecoinvent - Wastewater treatment from organic chemical production)

2. PNIPAM (Poly(N-isopropylacrylamide))

Synthesized by free radical polymerization of NIPAM monomer derived from fossil precursors (acryloyl chloride and isopropylamine).

Step 1: Monomer Production (NIPAM)

Synthetic route involves multi-step reaction (Acrylonitrile $\rightarrow$ Acryloyl Chloride + Isopropylamine). Monomer requirement: $1.02\text{ kg NIPAM/kg polymer}$.

Monomer GHG intensity: $1.02 \times 4.80\text{ kg CO}_2\text{e/kg} = 4.90\text{ kg CO}_2\text{e/kg}$. (Data source: GaBi/Sphera Database & Literature LCA on specialty acrylamides)

Step 2: Biogenic Carbon Sequestration

$0.00\text{ kg CO}_2\text{e/kg}$ (100% fossil origin).

Step 3: Catalyst / Radical Initiator

Initiator (Azobisisobutyronitrile, AIBN / APS, ~0.01 kg/kg): $0.01 \times 3.50\text{ kg CO}_2\text{e/kg} = 0.04\text{ kg CO}_2\text{e/kg}$. (Data source: Ecoinvent - Fine chemicals production)

Step 4: Solvents & Purification Reagents

Polymerization solvent (e.g., dioxane, acetone, or solvent-precipitant mixtures like hexane/ether for purification). Even with recovery, downstream fractionation incurs losses (~0.35 kg makeup solvent/kg PNIPAM): $0.35 \times 2.20\text{ kg CO}_2\text{e/kg} = 0.77\text{ kg CO}_2\text{e/kg}$. (Data source: Ecoinvent - Organic solvents)

Step 5: Process Energy (Polymerization, Vacuum Drying, Lyophilization)

Free radical polymerization operates at lower temperatures (30–60°C), but downstream precipitation, drying, or lyophilization is energy-intensive.

Electricity: $1.8\text{ kWh/kg} \times 0.45\text{ kg CO}_2\text{e/kWh} = 0.81\text{ kg CO}_2\text{e/kg}$.

Thermal: $4\text{ MJ/kg} \times 0.06\text{ kg CO}_2\text{e/MJ} = 0.24\text{ kg CO}_2\text{e/kg}$.

Step 6: Waste Stream Treatment

Hazardous organic waste incineration (spent wash liquors, oligomer residuals): $0.30\text{ kg organic waste} \times 1.65\text{ kg CO}_2/\text{kg} = 0.50\text{ kg CO}_2\text{e/kg}$. (Data source: US EPA Waste Reduction Model / Ecoinvent hazardous waste incineration)

3. Bio-PA (Itaconic Acid & Cadaverine) — Experimental / Lab Scale

Lab-scale synthesis relies on batch precipitation of the nylon salt using ethanol, followed by multi-stage thermal polycondensation in a laboratory vacuum oven.

Step 1: Monomer Production (Bio-routes)

Itaconic Acid (IA, via fungal glucose fermentation, ~0.56 kg/kg PA): $0.56 \times 0.90\text{ kg CO}_2\text{e/kg} = 0.50\text{ kg CO}_2\text{e}$. (Data source: Nature Sustainability bio-itaconic LCA / Ecoinvent)

Cadaverine (1,5-diaminopentane, via engineered bacterial decarboxylation of bio-lysine, ~0.44 kg/kg PA): $0.44 \times 1.45\text{ kg CO}_2\text{e/kg} = 0.64\text{ kg CO}_2\text{e}$. (Data source: Bioresource Technology - Bio-diamines life cycle)

Subtotal Monomers: $1.14\text{ kg CO}_2\text{e/kg}$

Step 2: Biogenic Carbon Sequestration

Polyamide contains $\sim 57.6%$ biogenic carbon derived from biological atmospheric fixation:

Stoichiometry: $1\text{ kg PA} \times 0.576\text{ kg C} \times (44/12) = -2.11\text{ kg CO}_2\text{e/kg}$. (Data source: GHG Protocol Product Life Cycle Standard - Biogenic Removals)

Step 3: Catalyst / Additives

Phosphoric/phosphite catalyst or thermal stabilizer (~0.005 kg/kg): $0.005 \times 2.80\text{ kg CO}_2\text{e/kg} = 0.01\text{ kg CO}_2\text{e/kg}$.

Step 4: Solvents (Lab-scale Ethanol Salt Formation)

Lab-scale precipitation and washing uses high ethanol-to-monomer ratios. With imperfect lab rotary evaporator recovery (~75% recovery, 25% loss): $0.60\text{ kg loss} \times 1.50\text{ kg CO}_2\text{e/kg} = 0.90\text{ kg CO}_2\text{e/kg}$. (Data source: Ecoinvent - Bioethanol / synthetic ethanol)

Step 5: Process Energy (Lab-Scale Electric Ovens & High Vacuum)

Salt drying and pre-polymerization ($110^\circ\text{C}$ for 24h) in low-capacity ovens: $3.2\text{ kWh/kg}$.

Final high-temp melt polymerization ($180^\circ\text{C}$ for 2h under high vacuum): $1.4\text{ kWh/kg}$.

Total lab power: $4.6\text{ kWh/kg} \times 0.45\text{ kg CO}_2\text{e/kWh} = 2.07\text{ kg CO}_2\text{e/kg}$.

Step 6: Lab Waste & Rinsing Disposal

Laboratory organic solvent disposal / incinerated waste streams: $0.35\text{ kg CO}_2\text{e/kg}$.

4. Bio-PA (Itaconic Acid & Cadaverine) — Industrial Scale

Industrial-scale optimization replaces alcoholic salt isolation with continuous concentrated aqueous nylon salt processes, heat recovery networks, and direct continuous melt polycondensation.

Step 1: Monomer Production (Industrial Scale Efficiencies)

Continuous high-cell-density fermentation, combined heat and power (CHP) integration:

Itaconic Acid ($0.56\text{ kg} \times 0.70\text{ kg CO}_2\text{e/kg} = 0.39\text{ kg CO}_2\text{e}$).

Cadaverine ($0.44\text{ kg} \times 1.10\text{ kg CO}_2\text{e/kg} = 0.48\text{ kg CO}_2\text{e}$).

Subtotal Monomers: $0.87\text{ kg CO}_2\text{e/kg}$. (Data source: Industrial biotechnology life cycle models / Cathay Biotech benchmarks)

Step 2: Biogenic Carbon Sequestration

Same stoichiometric incorporation: $\mathbf{-2.11\text{ kg CO}_2\text{e/kg}}$.

Step 3: Catalyst / Stabilizers

Micro-dosed antioxidant/catalyst (e.g., sodium hypophosphite, ~0.002 kg/kg): $0.01\text{ kg CO}_2\text{e/kg}$.

Step 4: Solvents

100% aqueous solution salt formulation (eliminates organic solvent requirement): $0.00\text{ kg CO}_2\text{e/kg}$.

Step 5: Process Energy (Autoclave Concentration, Melt Polycondensation, Extrusion)

Thermal pinch analysis and steam recompression reduce external heat inputs:

Process steam: $3.5\text{ MJ/kg} \times 0.06\text{ kg CO}_2\text{e/MJ} = 0.21\text{ kg CO}_2\text{e/kg}$.

Electricity (Agitation, vacuum pumps, pelletizing): $0.65\text{ kWh/kg} \times 0.45\text{ kg CO}_2\text{e/kWh} = 0.29\text{ kg CO}_2\text{e/kg}$.

Subtotal Energy: $0.50\text{ kg CO}_2\text{e/kg}$. (Data source: PlasticsEurope - Polyamide 6 / 66 industrial eco-profiles adjusted for bio-feedstocks)

Step 6: Industrial Wastewater & Waste Treatment

Condensate water of polycondensation containing trace diamine sent to biological WWTP: $0.08\text{ kg CO}_2\text{e/kg}$.

(Note: When full biogenic sequestration credit is included according to ISO 14067 / GHG Protocol Product Standard, industrial Bio-PA achieves a "cradle-to-gate" net-negative carbon balance. If biogenic uptake is excluded, gross cradle-to-gate emissions are $+1.46\text{ kg CO}_2\text{e/kg}$.)

**Summary Table: Cradle-to-Gate Life Cycle Breakdown ($\text{kg CO}_2\text{e}$ per kg Polymer)**

Data Sources:

Monomer Values: Derived from life cycle inventory (LCI) databases for bio-based chemicals and petrochemical precursors.

Energy Factors: Based on global average electricity grid emission factors (~0.45 kg CO2e/kWh).

Sequestration: Calculated based on the stoichiometric carbon content of the bio-based monomers.

Processing Data: Derived from experimental parameters provided (temperature/time) and industrial benchmarks for large-scale polycondensation.

**2. End-of-Life (EoL) Recyclability & Circularity Pathways**

The holistic sustainability of these polymers depends heavily on their end-of-life fate:

HPC (Hydroxypropyl Cellulose):

Biodegradation & Composting: Modified cellulose possesses moderate to high biodegradability depending on the Degree of Substitution (DS). Low-DS HPC breaks down readily in industrial and municipal wastewater aerobic treatment systems without toxic persistent residues.

Mechanical/Chemical Recycling: Not amenable to conventional mechanical melting due to thermal degradation before melting. Chemical recovery back to cellulose is economically non-viable. Best managed via organic diversion (composting) or industrial anaerobic digestion.

PNIPAM:

Recyclability Constraints: Because it is primarily synthesized as crosslinked hydrogels or functional linear polymers for targeted stimuli-responsive applications (e.g., drug delivery, sensors), PNIPAM cannot be mechanically recycled in commercial post-consumer sorting streams.

Chemical Depolymerization: Carbon-carbon backbones are chemically resilient, making catalytic cracking or chemical de-polymerization back to NIPAM energy-intensive and cost-prohibitive.

Disposal Fate: Primarily clinical or municipal solid waste incineration. Thermal destruction releases $\text{NO}_x$ and fossil $\text{CO}_2$, requiring advanced flue gas scrubbing.

Bio-PA (Polyamide from Itaconic Acid & Cadaverine):

Mechanical Recycling: As a thermoplastic polyamide, Bio-PA can be repeatedly re-melted, extruded, and compounded (similar to PA54 or PA56), fitting into existing mechanical nylon recycling streams.

Chemical Upcycling (Solvolysis/Hydrolysis): The amide linkages can undergo catalyzed acid/base hydrolysis or ammonolysis back into itaconic acid and cadaverine precursors at high selectivity, enabling true circular closed-loop recycling.

Carbon Durability: Unlike short-lived bioplastics, engineering polyamides used in durable goods retain biogenic carbon for long operational lifespans (10–20+ years), maximizing carbon storage impact.

**3. Techno-Economic Analysis (TEA) & Viability Assessment**

Feedstock Costs & Green Premium:

PNIPAM: Highly priced ($> $80 - $250/\text{kg}$ specialty grade). Driven by the multi-step synthetic complexity of NIPAM monomer and high purification costs. Production is geared toward ultra-high-margin biomedical niches where carbon footprint is currently a secondary purchasing factor.

HPC: Mature industrial commodity-specialty chemical ($$8 - $15/\text{kg}$). Cost volatility correlates with purified wood pulp and petrochemical propylene oxide prices. The infrastructure is fully amortized, meaning minimal Capex risk.

Bio-PA (Industrial vs. Experimental): Lab-scale routes are economically unfeasible ($>$100/\text{kg}$) due to manual ethanol salt precipitation and prolonged oven batch times. At commercial scale, bio-itaconic acid ($\sim $1.50 - $2.20/\text{kg}$) and bio-cadaverine ($\sim $2.50 - $3.20/\text{kg}$) bring polymer production costs to approximately $$2.80 - $4.00/\text{kg}$. While this represents a $20–40%$ green premium over fossil-based PA66 or PA6, it delivers parity with specialty engineering polyamides (e.g., PA11, PA12).

Process Scaling Bottlenecks for Bio-PA:

Thermal Stability of Itaconic Acid: The conjugated double bond in itaconic acid can undergo uncontrolled crosslinking, isomerization, or decarboxylation at elevated polycondensation temperatures ($>180^\circ\text{C}$). Industrial success requires specialized catalytic control or microwave/continuous-flow reactors to maintain high linear molecular weights without gelation.

Solvent-Free Salt Processing: Transitioning from solvent-based precipitation to concentrated water-based salts or reactive extrusion is the critical tipping point for both economic viability and achieving the projected negative net-carbon footprint.
